# Radical hemithorax radiotherapy induces an increase in circulating PD-1^+^ T lymphocytes and in the soluble levels of PD-L1 in malignant pleural mesothelioma patients: a possible synergy with PD-1/PD-L1 targeting treatment?

**DOI:** 10.3389/fimmu.2025.1534766

**Published:** 2025-04-01

**Authors:** Alberto Revelant, Francesca Gessoni, Marcella Montico, Raja Dhibi, Giulia Brisotto, Mariateresa Casarotto, Martina Zanchetta, Veronica Paduano, Filippo Sperti, Chiara Evangelista, Fabiana Giordari, Valli De Re, Marco Trovò, Emilio Minatel, Maurizio Mascarin, Agostino Steffan, Elena Muraro

**Affiliations:** ^1^ Division of Radiation Oncology, Centro di Riferimento Oncologico di Aviano (CRO), IRCCS, Aviano, Italy; ^2^ Clinical Trial Office, Scientific Direction, Centro di Riferimento Oncologico di Aviano (CRO), IRCCS, Aviano, Italy; ^3^ Immunopathology and Cancer Biomarkers Unit, Department of Translational Research, Centro di Riferimento Oncologico di Aviano (CRO), IRCCS, Aviano, Italy; ^4^ Biobank, Department of Translational Research, Centro di Riferimento Oncologico di Aviano (CRO), IRCCS, Aviano, Italy; ^5^ Department of Radiation Oncology, Udine General Hospital, Udine, Italy

**Keywords:** malignant pleural mesothelioma, radiotherapy, biomarkers, tcr, anti-tumor immunity

## Abstract

Malignant Pleural Mesothelioma (MPM) is an aggressive tumor associated with asbestos exposure, characterized by a poor prognosis, managed with surgery, chemotherapy and radiotherapy. Recently, immunotherapy gives a survival advantage compared to chemotherapy, but limited to the non-epithelioid histotype, the rarest type. Radical hemithorax radiotherapy (RHRT) improves the Overall Survival (OS) of MPM patients, irrespective of histotype, and is able to induce immunomodulatory effects. In this study we aim to investigate changes in circulating T lymphocytes phenotype and activity, in MPM patients undergoing RHRT, to evaluate a possible therapeutic space for immunotherapy in this setting. To assess immunomodulatory effects of RHRT we evaluate peripheral blood samples of 35 MPM patients collected before treatment, at the end of RT, and 1 month later. We first notice that higher Lymphocyte-to-Monocyte Ratio (LMR) levels, before RT, are associated with an improved OS. The immune monitoring performed by ELISA assays reveals a significant increase in the serum levels of soluble PD-L1 (sPD-L1) and IFN‐γ at the end of RHRT. Furthermore, the percentage of PD‐1^+^ cells, evaluated by flow cytometry, significantly raise after RHRT in T cells, both CD4^+^ and CD8^+^. Also the proportion of proliferative cells is significantly expanded after RHRT in all T cell subtypes. After treatment we observe a significant increase in the number of patients showing WT-1 specific CD4^+^ T cells, measured by intracellular staining. The TCR repertoire analysis, investigated by Next Generation Sequencing, reveals an increased number of expanded T-cell clones after RHRT, and an association between TCR clonality and the percentage of proliferating cytotoxic T lymphocytes. The comparison of TCR sequences obtained in our cohort with those described in a literature cohort of MPM patients, reveals common entries, specific for MPM-associated antigens including WT-1. In this setting, pre-treatment levels of LMR seem to have a positive prognostic role, and RHRT would appear to induce immunomodulating effects, potential biomarkers for immunotherapy eligibility: i.e. increased PD-1^+^ T lymphocytes, proliferating T cells, expanded T cell clones and augmented levels of sPD-L1. These data suggest the design of a prospective study evaluating a maintenance immunotherapy after RHRT in MPM, even in the epithelioid histotype.

## 1 Introduction

Malignant pleural mesothelioma (MPM) is a rare and aggressive neoplasm of the pleura with limited second-line treatment options. The development of MPM is closely associated with exposure to asbestos fibers. The incidence of MPM is generally higher in males than in females, primarily due to historical differences in asbestos exposure. Worldwide, the standardized incidence rates per 100,000 persons are 0.7 in males and 0.3 in females in the United States and 1.7 in males and 0.4 in females in Europe (1). In 2021, the World Health Organization (WHO) updated the histological classification of MPM. The current classification identifies three main histological subtypes: epithelioid, sarcomatoid, and biphasic mesothelioma (2, 3). In epithelioid histology, it is becoming increasingly important to assess the degree of cell differentiation (high grade or low grade) and the architectural pattern in order to accurately stratify the aggressiveness of the disease (2, 4). Treatment of MPM should be determined by a multidisciplinary team, taking into account factors such as disease stage, histological subtypes, patient age, Performance Status (PS), and patient preferences. Management options include surgery (SU), chemotherapy (ChT), immunotherapy (IT), and radiation therapy (RT). Surgical intervention may be useful to obtain a histological diagnosis and to control symptoms. In selected cases, in patients with good PS and disease localized to the pleura, it is possible to opt for a radical surgical approach combined with other treatments (SU combined with ChT and RT). In these patients, lung-sparing surgery is currently preferable to pneumonectomy (5–7). First-line systemic platinum-based doublet therapy should be considered for all MPM patients with epithelioid histology and good PS (PS, 0-2) (8, 9). Immune checkpoint inhibitors (ICIs) were also evaluated in first-line treatment as single agents. Some important clinical trials have investigated the combination of radiotherapy and immunotherapy in the treatment of solid tumours at different stages of the disease. In early-stage non-small cell lung cancer (NSCLC), two randomized (phase II) trials investigated the interaction between stereotactic body radiation therapy (SBRT) and immunotherapy drugs. The Chang et al. study is a randomized trial evaluating SBRT with or without concurrent or adjuvant durvalumab in patients with stage Ia to IIb NSCLC. The study showed a significant event-free survival benefit in favour of the durvalumab arm (77% versus 53% at four years) (10). Another randomized trial included patients with stage I to IIIa NSCLC. These received neoadjuvant durvalumab with or without SBRT. This study showed an increase in pathological complete response in 53% of patients who had received the combination treatment compared to 7% who had received durvalumab alone (11). However, in locally advanced thoracic oncology, the main trial with positive results is PACIFIC. This study examined concomitant chemo-radiotherapy treatment followed or not by durvalumab in patients with inoperable NSCLC. The study showed stable progression-free survival (PFS) and overall survival (OS) benefits in the durvalumab arm. This trial changed the treatment of the disease and became the standard of care (12). For metastatic disease, research is underway to determine which patient populations may benefit from the combination of radiotherapy and immunotherapy drugs, which doses of radiotherapy are most effective, and the number and location of lesions that can be safely treated (13). In the MPM setting, the phase III study Check Mate-743 randomized 605 patients with unresectable MPM of both histologies (epithelioid and non-epithelioid) to the combination of nivolumab plus ipilimumab for two years versus six cycles of cisplatin-pemetrexed or carboplatin-pemetrexed. The study concluded with a significant increase in OS in patients with non-epithelioid MPM treated with nivolumab-ipilimumab. This advantage appears limited in epithelioid histology with minimal benefit versus conventional ChT (14). Ipilimumab-nivolumab is currently a first-line treatment option in non-epithelioid histology approved by the Food and Drug Administration (FDA) and the European Medicines Agency (EMA). PD-L1 is expressed in 40%-60% of MPM tumors cells, particularly in sarcomatoid subtypes (15). In several studies, PD-L1 expression was weakly correlated with the response to ICIs, either alone or in combination with CTLA-4 inhibitors. This was also observed in the preliminary analysis of CheckMate 743, where OS with ICIs was better than with ChT in PD-L1-positive MPM, but not in PD-L1-negative cases (16–18). However, there is currently limited evidence regarding the efficacy of both ChT and IT as second-line treatment. A number of trials evaluating ICIs have been conducted in second or subsequent line settings, using agents targeting CTLA-4, PD-1 and PD-L1. The DETERMINE study compared tremelimumab with placebo and showed no OS benefit (16). The PROMISE-meso randomized phase III clinical trial compared pembrolizumab with second-line vinorelbine or gemcitabine monotherapy, and demonstrated no OS benefit (19).

RT also plays an important role in other settings like adjuvant, neoadjuvant or palliative therapy. For limited disease RT is delivered after surgery to the entire pleural space at a dose of 50-60 Gy in conventional fractionation. The feasibility and safety of these treatments have been evaluated in multiple clinical trials (20–25). Ionizing radiation (IR) interacts with the tumor microenvironment (TME) and modulates the immune system (26). IR causes acute cellular damage resulting in the development of acute inflammation, leading to changes in the TME, increased chemokine production and increased tissue infiltration by T lymphocytes (27). IR induces the expression of major histocompatibility complex molecules in tumor cells, which in turn leads to an increase in the presentation of pre-existing antigens or neoantigens resulting from DNA damage (28). At the same time, however, IR induces suppression of anti-tumor immunity by recruiting regulatory T lymphocytes (Treg), macrophages and myeloid-derived cells, all of which exert an immunosuppressive activity (29). Tregs are a subpopulation of CD4^+^ T cells characterised by forkhead box P3 (FoxP3) expression and high levels of CD25. Treg exerts undesired immunosuppressive effects and may promote tumour progression or the development of metastases (30). There is a general consensus that Tregs are immunosuppressive and may contribute to treatment failure. The effects of IR on Tregs in the TME are complex. It has been reported that IR can increase the recruitment of Tregs into the TME in various cancers, which may contribute to radioresistance. IR significantly increases tumour-infiltrating Tregs in several murine tumour models (30–32). A previous study conducted at our institute (33) aimed to evaluate the immunomodulatory effects of Radical Hemithoracic RadioTherapy (RHRT) in patients with MPM. The study observed immune changes, such as an increase in activated T cells and Interferon (IFN)-γ-producing T helper 1 (Th) cells, following RHRT. In addition, increased basal levels of Th22 and Interleukin (IL)-10 and increased T cells were associated with improved survival in patients who underwent radical RT (33). T lymphocytes, particularly CD8^+^ T lymphocytes activated by antigen-presenting cells such as dendritic cells, play a key role in IR-induced anti-tumor activity. IR increases peritumoral lymphocyte infiltration and enhances T-cell cytotoxicity by increasing the production of Tumor Necrosis Factor (TNF)-α and IFN-γ. However, this type of response tends to be rapidly exhausted and limited by immune checkpoints in the TME such as PD-L1 and CTLA-4 (34). In this study we investigated the changes in circulating T lymphocyte populations in patients with MPM who are undergoing RHRT. The aim was to evaluate, in these patients, a possible future therapeutic space for immunotherapy associated with this type of RT.

## 2 Materials and Methods

### 2.1 Patients enrollment

Immunomodulatory effects of RHRT were assessed in peripheral blood samples from 35 MPM patients treated between July 2020 and August 2022. The clinical study design is summarized in **Supplementary Figure S1**. Inclusion criteria encompassed individuals aged ≥ 18 years with a histologically confirmed diagnosis of MPM, non-radical lung-sparing surgery, evident gross residual disease post-surgery, stage I-IVA (TNM stage 7th edition), an ECOG PS score of 0-2, pulmonary function of at least 50%, prior platinum/pemetrexed doublet ChT, technical feasibility for RHRT and satisfactory bone marrow function (white blood cells [WBC] ≥ 2e9/L, platelets [PLT] ≥ 10e9/L, hemoglobin [Hb] > 100 g/L). Exclusion criteria included pathological contralateral mediastinal lymph nodes (N3), metastatic MPM (stage IVb), or intra-fissural disease. Tumor histology was classified as epithelioid or non-epithelioid (sarcomatoid and biphasic). Staging was performed using contrast-enhanced computed tomography (CT) scans of the lungs and abdomen, along with 18F-fluorodeoxyglucose (18F-FDG) positron emission tomography (PET)/CT.

All patients underwent RHRT, with the Planning Target Volume (PTV) meticulously delineated to include the entire thoracic thickness, intercostal muscles, preoperative pleural surface, chest wall, and surgical scars, while excluding the interlobar pleura. Superficial surgical scars were not corrected by bolus positioning.

A 3 to 4 mm margin was added at the lung-rib interface to ensure comprehensive pleural coverage. The superior PTV boundary extended 1.5 cm above the lung apex, incorporating a portion of the infraclavicular fossa. The posterolateral and anterolateral borders of the chest wall overlapped the posterior vertebral bodies and the anterior sternum, respectively. Medially, the PTV included the ipsilateral pericardium, and in cases with pathologic ipsilateral mediastinal nodules (N1-2), these nodes were encompassed within the PTV. The lower PTV boundary included the entire diaphragmatic dome up to the insertion of the diaphragm bone, typically reaching the level of the L2 vertebral body. A total dose of 50 Gy was delivered in 25 fractions with a prescribed isodose of 95% to the PTV and a simultaneous integrated boost technique of 60 Gy to the Gross Tumor Volume (GTV). Suspected disease progression was evaluated using 18F-FDG PET/CT, which was routinely performed every 6 months.

OS was defined as the time from the first day of RHRT to death from any cause, last follow-up or the reach of 24 months of follow-up (April 2024). Progression-free survival (PFS) was defined as the time from the first day of RHRT to death and either local or distant failure.

This study adhered to the ethical principles outlined in the Declaration of Helsinki, received approval from the local ethical committee (Comitato Etico Unico Regionale [CEUR], CRO-2023-63, MESORTIBO study), and was published on clinicaltrials.gov (NCT06637345). Additionally, a retrospective analysis was conducted on 43 MPM patients enrolled in a randomized phase III trial from August 2014 to June 2017 which was also approved by the local Ethics Committee (Comitato Etico Indipendente del CRO di Aviano, CRO-2013-38). Patients were randomized to receive RHRT treatment (interventional arm; n=21) or palliative RT (control arm, n=22). The target volume for RT included surgical scars and/or gross residual disease identified by PET/CT, and radiation dose schedules varied from 21 Gy in 3 fractions to 20-30 Gy in 5-10 fractions delivered 5 days per week (35). RT techniques used were conventional RT, 3-D conformal RT, and/or electrons. Written informed consent was obtained from all participants.

### 2.2 Sample collection and blood count

Blood and serum samples were collected from patients before treatment (baseline), at the end of RT treatment, and 1 month after the end of RT treatment, and transported at room temperature. Samples were managed by the Easy Track (Twin Helix) program, which maintains correct traceability of the samples and their related data. Blood count was routinely performed with a XN-3100TM automated hematology analyzer (Sysmex). Neutrophil-to-Lymphocyte Ratio (NLR) was calculated as the total neutrophil count (10e9/L) divided by the total lymphocyte count (10e9/L), Lymphocyte-to-Monocyte Ratio (LMR) as total lymphocyte count (10e9/L) divided by the total monocyte count (10e9/L), Systemic Immune-inflammation Index (SII) as neutrophil count (10e9/L) x platelet count (10e9/L) divided by lymphocyte count (10e9/L). Peripheral Blood Mononuclear Cells (PBMCs) were freshly isolated (within 4 h after blood draw) from blood in EDTA-tube by Ficoll-Hypaque gradient (Lymphoprep, Fresenius Kabi Norge Halden) using standard gradient separation. The cells were washed in Phosphate Buffered Saline (PBS; Lonza), counted using an automatic cell counter (ADAM, Twin Helix; viability >95%) and viably frozen in a solution of 90% heat-inactivated Fetal Bovine Serum (FBS; Euroclone) and 10% DMSO at −80°C for 24 h, then stored in liquid nitrogen until use. After thawing in RPMI (Lonza) containing 2 mM L-glutamine, 100 µg/mL streptomycin, and 100 IU/mL penicillin (Sigma-Aldrich), supplemented with 10% FBS (Sigma-Aldrich), cells were washed in PBS and counted again to check viability (cell viability required >85%). Serum samples were processed by centrifugation at 3600 rpm for 10 minutes at room temperature, then aliquoted into four 500 µL Matrix tubes (Thermo Scientific), placed in a specific box and stored at -80°C until use.

### 2.3 T cells primary culture

Spontaneous T-cell responses against known MPM-associated antigens were evaluated in pre-stimulated patients’ PBMCs (36), obtained from the cohorts of patients included in the randomized trial described above. This analysis was not performed on samples obtained from patients enrolled between July 2020 and August 2022 due to limited available biological material. Briefly, thawed PBMCs were cultured in T-cell medium (IMDM containing 2 mM L-glutamine, 100 μg/mL streptomycin and 100 IU/mL penicillin, supplemented with 10% human serum) in the presence of 5 ng/mL Interleukin (IL)-7 (PromoKine) and IL-4 (PromoKine), at 37°C and 7.5% of CO_2_. One day after thawing, cells were stimulated with 1 μg/mL Human Leukocyte Antigen (HLA)-matched peptides, from MPM-associated antigens (mesothelin and Wilms Tumor-1 [WT-1]) or viral controls [Human Immunodeficiency Virus (HIV), Flu, Cytomegalovirus (CMV), Epstein Barr Virus (EBV)], supplemented with 5 ng/mL IL-7 and IL-4, and cultured at 37°C and 7.5% CO_2_. Selected epitopes with the corresponding viral/tumor antigen, HLA restriction, and reference are listed in the Supplemental Material (**Supplementary Table S1**). Cells were supplemented with 2 ng/mL IL-2 (PromoKine) every 2 days. After 12 days, pre-stimulated cells were collected, counted and stimulated overnight with individual peptides, in the presence of α–CD107a FITC (**Supplementary Table S1**), Golgi-STOP solution (protein transport inhibitor containing monensin, BD Biosciences), and 10 μg/mL Brefeldin (Sigma-Aldrich) at 37°C and 7.5% CO_2_. Non-specific stimulation with 5 ng/mL Phorbol 12-Myristate 13-Acetate (PMA, Sigma-Aldrich) and 1 μg/mL Ionomycin (Sigma-Aldrich) was used as control for cytokine production.

### 2.4 Flow cytometry

The antibodies used for flow cytometry are listed in **Supplementary Table S2**. LIVE/DEAD^®^ Fixable Aqua Dead Cell Stain kit (Molecular Probes, Thermo Fisher Scientific) was used to determine cell viability. Properly labeled isotypic antibodies were used as negative controls. All antibodies were diluted in an appropriate volume of 2% FCS in PBS to reduce non-specific signal and re-suspended in an appropriate volume of 1% paraformaldehyde in PBS. Intracellular FoxP3 and Ki-67 were determined using the eBioscience FoxP3 Staining Buffer Set (eBioscience), according to the manufacturer’s instructions. Briefly, after surface molecules staining, cells were fixed and permeabilized with fixation/permeabilization buffer for 30 min at 4°C, washed twice, and labeled with FoxP3 and Ki-67 antibodies in the presence of the permeabilization buffer at 4°C for at least 30 min, and after two additional washes, cells were re-suspended in PBS. To characterize Th1, Th17, and Th22 cells, PBMCs were pretreated with 50 ng/mL PMA (Sigma-Aldrich) and 1 µg/mL ionomycin (Sigma-Aldrich) in the presence of Golgi-STOP solution (a protein transport inhibitor containing monensin, BD Biosciences) and 10 µg/mL Brefeldin (Sigma-Aldrich) in T cell medium for 4 h at 37°C. To evaluate the production of IL-17, IL-22, Tumor Necrosis Factor (TNF)-α, Interferon (IFN)-γ, IL-2, and Macrophage Inflammatory Protein-1β (MIP-1β), cells were first stained for surface molecules, then fixed and permeabilized with the Cytofix/Cytoperm™ solution (BD Biosciences) for 20 min at 4°C. Following washing in PBS containing 0.5% Bovine Serum Albumin (BSA; Sigma-Aldrich) and 0.1% saponin (Sigma-Aldrich), cells were stained with antibodies in PBS + 0.5% BSA + 0.1% saponin at 4°C for 20 min. Samples were washed twice and re-suspended in PBS for flow cytometry analysis. At least 5x10e5 events were acquired. Flow cytometry analysis was performed with an LSR-Fortessa™ (Becton Dickinson) belonging to the flow cytometry core facility of our Institute. Photomultiplier voltages and compensation were set up with unstained and stained cells or with the CompBeads Set Anti-Mouse Ig or Anti-Rat Ig, k Sets (BD Biosciences). Flow cytometry data were analyzed with DIVA (BD) and FlowJo (Tree Star, Ashland, OR, USA) software, boolean gating analysis for intracellular staining was performed with SPICE 6 software (37). The production of cytokines after stimulation with MPM-associated antigens (mesothelin, MSTL; and Wilms Tumor-1, WT-1) was considered positive if the percentage of cytokine-positive cells at least doubled that observed after stimulation with negative controls (HIV-derived peptides, considered as non-specific signal). A cut-off of 0.01% cytokine-positive cells among CD8^+^ or CD4^+^ cells was used to define a positive population.Immune cell subsets were identified as follows: T cells as CD3^+^ lymphocytes, CD4 and CD8 T cells as CD3^+^CD4^+^CD8^-^ and CD3^+^CD4^-^CD8^+^ lymphocytes, inhibited CD3, CD4 or CD8 T cells as CD3^+^PD-1^+^, CD3^+^CD4^+^PD-1^+^ or CD3^+^CD8^+^PD-1^+^ lymphocytes, proliferating CD3, CD4 or CD8 T cells as CD3^+^Ki-67^+^, CD3^+^CD4^+^Ki-67^+^ or CD3^+^CD8^+^Ki-67^+^ lymphocytes, Treg as CD3^+^CD4^+^CD8^-^CD25^high^CD127^low^FoxP3^+^ lymphocytes (inhibited if expressing the PD-1 molecule, proliferating if showing a Ki-67 positive signal). IFN-γ- or TNF-α-producing CD3^+^CD4^+^ lymphocytes were classified as Th1 cells and IL-17- or IL-22-producing CD3^+^CD4^+^ lymphocytes were classified as Th17 or Th22 cells. An example of the gating strategy used to identify these cell populations was reported in **Supplementary Figure S2**.

### 2.5 ELISA

Serum levels of IL-6, IL-8, IL-10, TNF-α, and IFN-γ were evaluated using the Q-Plex™ Array Human Cytokine Panel 2 (Quansys Biosciences, TEMA Ricerca, Bologna, Italy), according to the manufacturer’s instructions. Briefly, a 6-plex array containing pre-spotted cytokine-specific antibodies was used. Standards and pre-diluted (1:2) samples were added in duplicate. After 1 h of incubation at room temperature and three washes, the Detection Mix was added to each well. After another 1 h incubation at room temperature and three washes, Stabilizing Solution was added to stabilize the signal. The addition of Streptavidin-HRP 1X, Substrate A and B+, and the acquisition of luminescent signal with the Q-View Imager LS, together with data analysis and processing through the Q-View Software, were performed by TEMA Ricerca laboratories’ customer service (Bologna, Italy).

Soluble Programmed Death-Ligand 1 (sPD-L1) serum levels were assessed through Quantikine^®^ ELISA (R&D Systems, Biotechne, Minneapolis, USA) under manufacturer’s instructions. Briefly, standards, controls, and samples were dispensed into the wells and the plate was incubated 2 h at room temperature, shaking. After four washes, human B7-H1 conjugate was added and incubated for 2 h at room temperature. After four more washes, Substrate Solution was added to wells, and the plate was incubated 30 minutes. The reaction was stopped and the absorbance was determined at 450 ± 10 nm using a microtiter plate reader (Infinite F200, Tecan, Switzerland) within 30 minutes.

### 2.6 Molecular analysis: T-Cell Receptor (TCR) sequencing and HLA typing

For TCR sequencing, genomic DNA was extracted from frozen PBMCs (ranging from 1.7 to 13.7x10e6 cells per sample) using the QIAamp DNA Blood kit (Qiagen) according to the manufacturer’s protocol. The concentration and quality of isolated DNA were assessed using a NanoDrop 2000c spectrophotometer (Thermo Fisher Scientific, USA). The extracted DNA was used for the deep resolution sequencing of the CDR3 regions of human TCR-β chains with the ImmunoSEQ hsTCRB_v4b Service (Adaptive Biotechnologies, Seattle, Washington, USA). Processed data were accessed for further analysis throughout the ImmunoSEQ Analyzer 3.0 software from Adaptive Biotechnologies. The ggplot package in R (version 4.4.1) was used to create the graph of the clones significantly expanded or contracted before and after RHRT. To investigate the specificity of expanded T cell clones, we used the tidyverse package in R to compare our patient CDR3 amino-acid sequences with those of 119 healthy donors. These sequences were downloaded from the Adaptive Biotechnologies’ immuneACCESS database and belong to the cohort 2 from a project that studied the impact of CMV on the TCR repertoire (38). The TCR sequences selected from our cohort were compared with those of each healthy donor, based on their CDR3 amino-acid sequences. The outputs containing common sequences present at least 5 times in healthy donors, along with their respective frequencies, were combined together.

HLA genotyping was performed through two different methods in the two cohorts of patients. Patients enrolled in the MESORTIBO study and treated between 2020 and 2022 were characterized using PCR-sequence-based typing (PCR-SBT), which targeted the exon 2 and 3 of HLA-A and HLA-B with primers specific for each class I locus as previously reported (39). PCR products were sequenced on the Applied Biosystems™ 3500 Dx Genetic Analyzer automated sequencer (Applied Biosystems™, Foster City, CA, USA). The sequences were assembled in pairs and identified with the Sequence Pilot software (JSI medical systems, Germany). The HLA Evolutionary Divergence (HED) Index was calculated through the HLA Evolutionary Divergence online tool (https://hladiv.net/) (40, 41). For the patients included in the randomized phase III study, samples were genotyped to identify those expressing the alleles HLA -A*02, -DRB1*01, -DRB1*03, -DRB1*04, and –DRB1*15 by performing PCR sequencing based typing with specific primers (42, 43). HLA background is reported in Supplemental Material (**Supplementary Tables S3, S4**).

### 2.7 Statistical Analysis

Data obtained from each parameter were expressed as median and interquartile range (IQR) and represented with a box plot, in which the whiskers indicate the lowest and the highest values included in the following interval: 1^st^ quartile - 1.5x(3^rd^-1^st^ quartile) and 3^rd^ quartile + 1.5x(3^rd^ -1^st^ quartile); values outside this interval are considered outliers (dots). Raw data can be provided by request. Normality assumption was evaluated both visually and with Shapiro-Wilk test to select the most appropriate parametric or non parametric test. For association analysis, the global cohort of patients was divided into two groups based on the median value of selected immune parameters measured at baseline. Patients were categorized into subgroups with values above (>p50, 50^th^ percentile) or below (≤p50) the median. OS, PFS, and Disease-Specific Survival (DSS) were estimated using the Kaplan–Meier method (the Log-rank test was used to compare the survival curves) and the Cox regression analysis, starting from the first day of the radiotherapeutic treatment to the event of interest or the last available follow-up. The non-parametric Friedman test was used to test differences between data collected at different time points. *Post-hoc* tests were carried out using Wilcoxon signed-rank test with Bonferroni correction for multiple (n=21) testing. Differences in the presence of T cell responses specific for MPM-associated antigens before and after RHRT were calculated based on chi-squared test. For TCR analysis the Morisita’s overlap index was calculated to determine similarities between samples, ranging from 0, as minimal, and 1, as maximal similarity. Student’s t test for two tailed distribution and unpaired data was employed to compare Morisita similarity index evaluated on samples from the same patient versus unmatched samples. The TCR clonality was evaluated through the Productive Simpson Clonality, which in turn is calculated as the square root of Simpson’s diversity index (a measure of diversity that takes into account the number of TCR clones identified in each sample and the relative abundance of each clone) for all productive rearrangements (unique TCR sequence that are able to encode a functional protein). Values range between 0, representing a polyclonal population, to 1, a monoclonal sample. The TCR richness was instead expressed as total productive templates, meaning those rearrangements that can produce a functional protein receptor (in frame and not containing a stop codon) and TCR entropy, calculated summing frequency times the log (base 2) of the same frequency over all rearrangements in a sample (higher entropy means a greater diversity of rearrangements). Differences between TCR entropy and total productive templates measured before and after RHRT were evaluated through the Grouped Comparison tool of the ImmunoSEQ Analyzer 3.0 software, which performed a Dunn’s test. The presence of expanded or contracted clones between paired samples was verified through the Differential abundance tool of the ImmunoSEQ Analyzer 3.0 software, and then compared through the Student’s t test for two tailed distributions and paired data. Differences between groups obtained by dichotomizing patients based on clinical parameters were evaluated through the Grouped Comparison tool of the ImmunoSEQ Analyzer 3.0 software. The non parametric Spearman’s rank correlation coefficient was calculated to find out possible correlations among the parameters measured after RHRT. The difference between the frequency of SARS-CoV-2 associated templates and the 20 most abundant templates measured after RHRT was evaluated through the Mann-Whitney U-test. In all cases, statistical significance was considered for p ≤ 0.05. By applying Bonferroni correction to the analysis of 21 parameters monitored throughout treatment, significance was considered for p<0.002.

## 3 Results

### 3.1 Patients characteristics and systemic inflammatory indexes

This study included a total of 35 patients with MPM. All characteristics of these subjects are summarized in **Table 1**. All patients were in good clinical condition (PS, 0-1) and underwent RHRT at a total dose of 50 Gy in 25 fractions. Six of these patients received a simultaneous integrated boost (SIB) to 60 Gy on PET-positive areas and macroscopic residual disease. Both epithelial (n=28) and non-epithelial (n=7) histologies were analyzed. Before RT treatment, patients underwent surgical procedures. Sixteen patients underwent thoracoscopic biopsy only, 3 underwent decortication, and 15 underwent pleurectomy/decortication. Only one case underwent pneumonectomy (EPP). All 35 patients also received systemic chemotherapy for a median of 4 cycles with cisplatin and pemetrexed. Regardless of the type of surgery, all patients presented with residual disease, either R1 (microscopic) or R2 (macroscopic). During follow-up, the observed median OS was 17 months (IQR, 10-36 months), while PFS and DSS were 10 months (IQR, 7-22 months) and 15 months (IQR, 10-36 months), respectively.

**Table 1:** Patients and tumor characteristics.

Clinical Parameter	RHRT-treated patients *(n=35)*
Age (median,min-max)	71 (48-88)
Sex (n, %)	
Male	28 (80)
Female	7 (20)
Histology (n, %)	
Epithelioid	28 (80)
Non-epithelioid	7 (20)
Surgery (n, %)	
Biopsy	16 (46)
Decortication	3 (8)
Pleurectomy/decortication	15 (43)
EPP	1 (3)

EPP, pneumonectomy; RHRT, radical hemithoracic radiation therapy.

**Figure 1:**
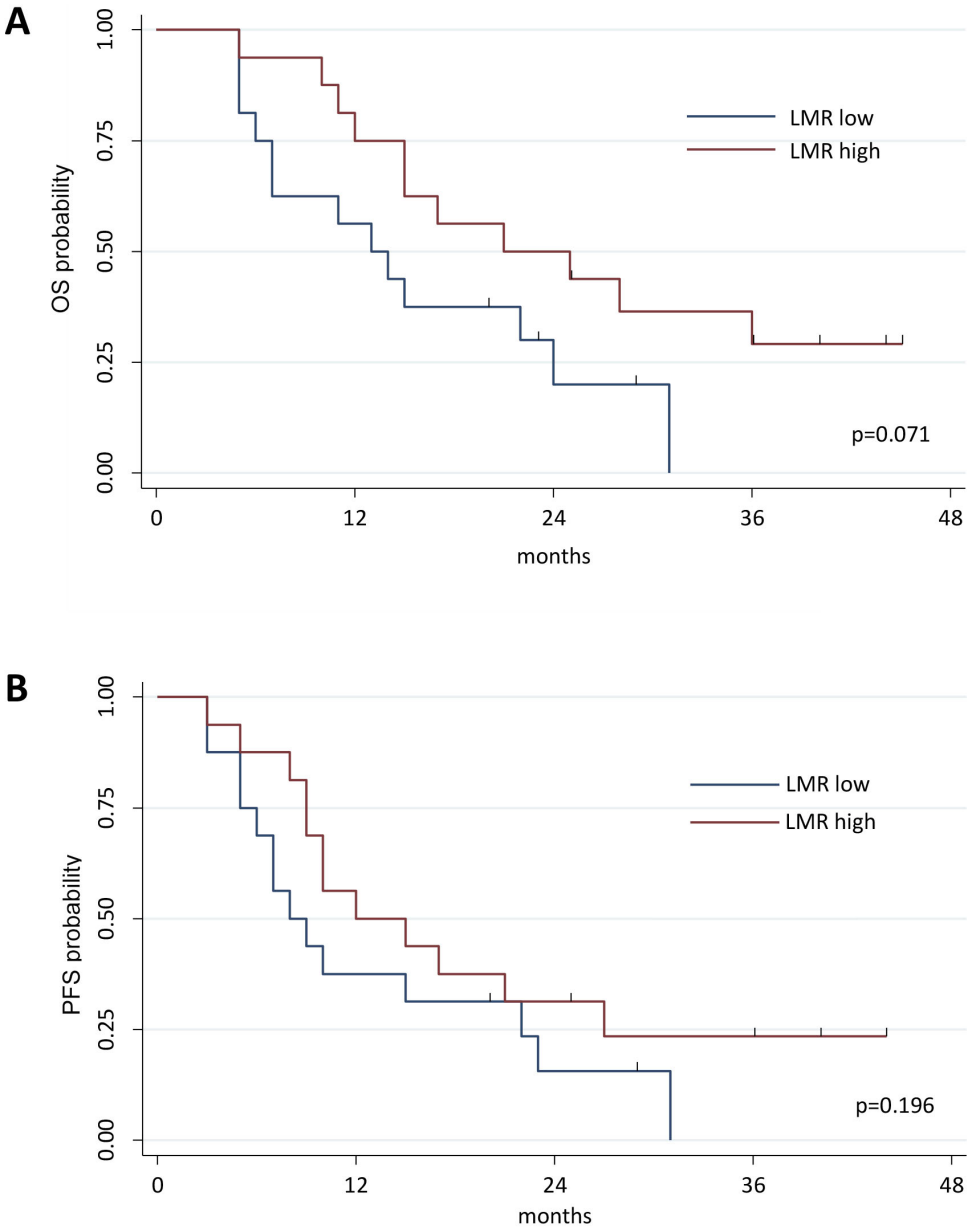
Kaplan-Meier curve estimates of Overall Survival (OS). Patients were divided into 2 groups based on the median value of LMR measured at baseline in the global cohort of patients; patients characterized by a LMR value above median value were classified as LMR high, whereas those with LMR value under the LMR median value were grouped as LMR low patients **(A)**. Evaluation of OS in the groups of patients characterized by a LMR value above or under LMR median value (Log-rank test, p=0.071). **(B)** Evaluation of PFS in the groups of patients characterized by a LMR value above or under LMR median value (Log-rank test, p=0.1961). OS, Overall Survival; PFS, Progression Free Survival.

Our primary objective was to evaluate the effects of RT on the immune landscape and explore how these interactions might influence treatment outcomes and inform the development of future targeted therapies. To achieve this, we first utilized the full blood count parameters collected before and after radiotherapy. In particular, we calculated the LMR, the NLR, and the SII. Patients were dichotomized based on LMR, NLR, and SII median values to assess a possible prognostic value of these indexes. A higher LMR value was associated with a better OS in Cox regression (HR 0.584, 95% CI 0.374-0.911; p=0.018), when dichotomized the LMR showed a better OS probability for value higher than the median, however this relation was not significant (Kaplan Meier analysis reported in **Figure 1A**, Log-rank test, p=0.071). No differences were found for PFS (Cox regression analysis HR 0.673, 95% CI 0.452-1.001, p=0.051; Kaplan Meier analysis reported in **Figure 1B**, log-rank test, p=0.196), nor for NLR and SII analyses (**Supplementary Table S5**). Analyses performed on indexes calculated after RT did not show any statistically significant difference (**Supplementary Table S5**).

### 3.2 Immune profiling of MPM patients undergoing RHRT

**Figure 2:**
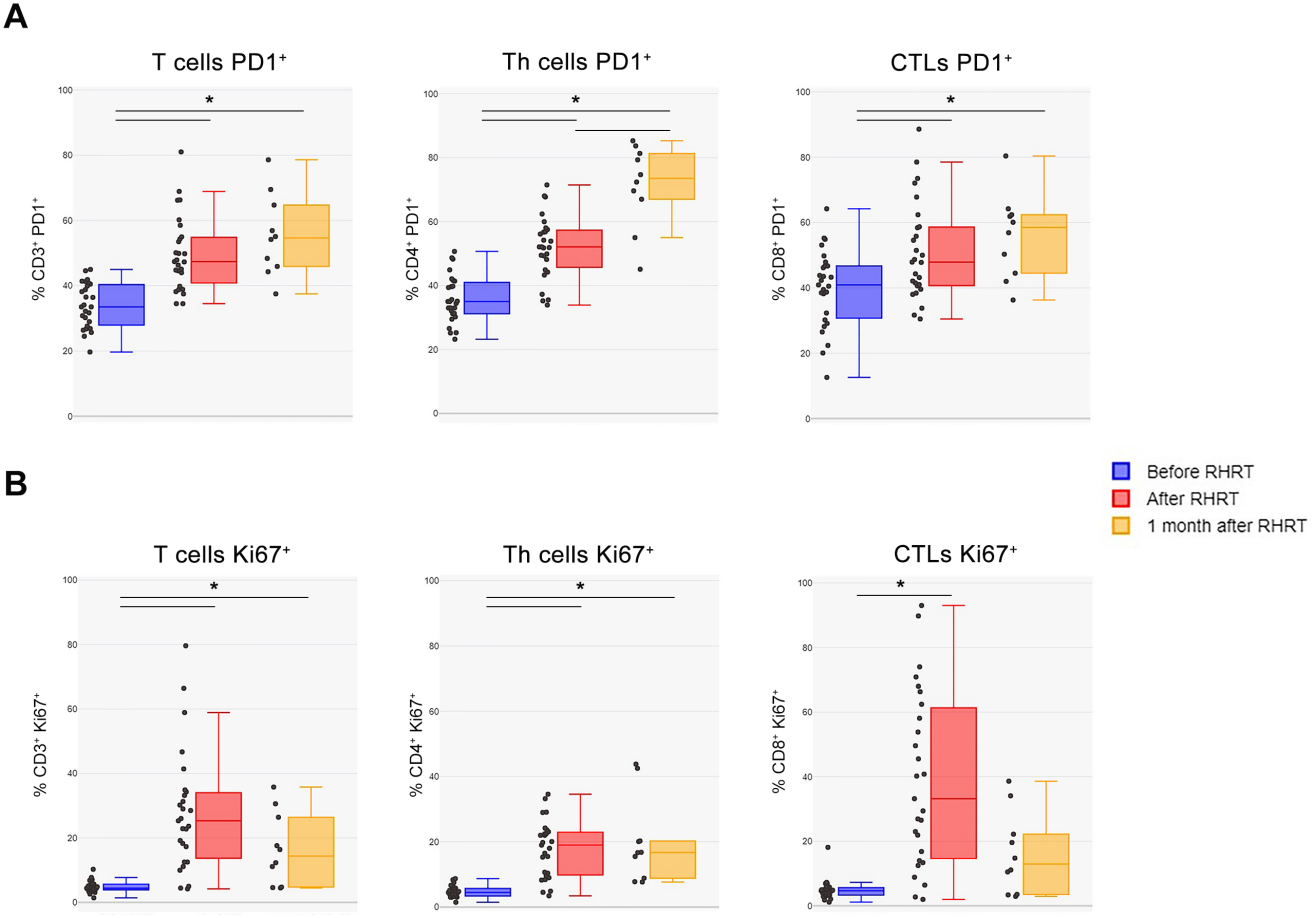
T cells characterization before and after RHRT. **(A)** The percentage of PD-1+ cells was evaluated in total T lymphocytes (CD3+ cells), in Th cells (CD3+CD4+ cells), and in CTLs (CD3+CD8+ cells) by using flow cytometry. **(B)** The proportion of proliferating cells was measured as the percentage of Ki67+ cells within total T cells (CD3+ cells), Th cells (CD3+CD4+ cells), and CTLs (CD3+CD8+ cells) by flow cytometry. Both analyses were performed in samples obtained before RHRT (n=27), at the end of RHRT (after RHRT; n=27), and 1 month after RHRT (n=10). Statistical differences were calculated by Wilcoxon test for paired data and considered significant if *p<0.002 (Bonferroni correction). CTLs, Cytotoxic T Lymphocytes; PD-1, Programmed Death 1; RHRT, Radical Hemithoracic Radiation Therapy; Th, T helper.

Our previous study performed in MPM patients enrolled in the phase III study, which demonstrated the survival advantage induced by RHRT over palliative RT (35), reported several transient and stable modulations in immune cell populations and cytokine levels, particularly in the arm of patients treated with RHRT (33). In the patients’ cohort enrolled for the present study we evaluated the same parameters before and after RHRT (n=35 for cytokines and soluble molecules, n=27 for immunophenotyping) and confirmed a slight decrease in IL-8 levels, an increase in IL-10 amounts, a reduction in the percentage of CD3^+^ T cells, and an increase in Treg and Th22 cells. However, only the variation in CD3^+^ T cells remained significant after Bonferroni correction (**Table 2**; gating strategy used for the identification of immune cell populations was reported in **Supplementary Figure S2**). Interestingly, when analyzing the levels of soluble PD-L1 (sPD-L1), we observed a significant increase of this Immune Checkpoint molecule after RHRT (**Table 2**). Moreover, as we previously noted that a change in T cell percentage after RHRT was associated with OS, we further characterized several parameters involved in the T-cell response (33). Notably, at the end of RHRT we observed higher levels of soluble IFN-γ than pre-treatment levels (**Table 2**). Furthermore, both the percentage of PD-1^+^ cells and the level of PD-1 expression significantly augmented after RHRT in all the investigated T cell subpopulations: global T cells (CD3^+^), T helper lymphocytes (Th, CD4^+^), cytotoxic T cells (CTL, CD8^+^) (**Figure 2A**, **Supplementary Figure S3** and **Supplementary Table S6**). This boost was maintained even 1 month after RHRT compared to pre-treatment levels (n=10; **Figure 2A**), and in Th cells even beyond the value observed at the end of RHRT (**Figure 2A**, central panel). Interestingly, also the proportion of proliferative cells (Ki67^+^) significantly expanded after RHRT in all the aforementioned T cell subtypes, and this trend was maintained 1 month after RHRT for CD3^+^ and Th lymphocytes (**Figure 2B**). The increase in the percentage of proliferative cells was observed in both PD-1_+_ and PD-1_+_ cells. Notably, the amount of CTLs Ki67^+^PD-1_+_ remained significantly higher compared to pre-treatment levels also 1 month after RHRT (**Supplementary Table S7**). Overall, these data suggested that the RHRT treatment affected the T cell compartment by influencing the functional status of T lymphocytes.

**Table 2:** Monitoring of serum cytokines, soluble molecules, and circulating immune cells in MPM patients undergoing RHRT.

Parameters	Before RHRT *Median (IQR)*	After RHRT *Median (IQR)*	p-value
IL-6	12.3 pg/mL (10.2-15.8)	12.5 pg/mL (10.7-26.6)	0.136
IL-8	27.1 pg/mL (19.2-38.5)	26.6 pg/mL (18.7-31.1)	0.006 *
IL-10	25.8 pg/mL (23.6-30.5)	30.8 pg/mL (26.1-36.9)	0.030 *
IFN-γ	36.8 pg/mL (30.2-43.5)	53.7 pg/mL (36.8-87.6)	<0.001 **
TNF-α	35.3 pg/mL (29.8-46.3)	34.4 pg/mL (29.3-43.1)	0.451
sPD-L1	85.7 pg/mL (79.2-104.7)	109.3 pg/mL (99.5-117.8)	<0.001 **
T cells (CD3)	69.3% (61.8-79.4)	60.9% (39.0-66.8)	<0.001 **
Th cells (CD4)	66.6% (56.1-74.6)	67.4% (49.8-75.8)	0.991
Th cells (CD4) w/o Tregs	61.3% (49.3-70.3)	60.7% (44.8-70.8)	0.581
CTLs (CD8)	27.0% (18.6-32.0)	24.4% (16.4-38)	0.530
Tregs	5.5% (4.8-6.5)	6.1% (4.3-9.3)	0.013 *
CTL/Treg	5.0 (3.8-6.0)	3.9 (2.0-6.2)	0.155
Th1 IFN-γ	9.4% (4.9-17.3)	8.5% (3.2-14.6)	0.106
Th1 TNF-α	8.6% (2.6-27.6)	14.6% (1.2-32.6)	0.324
Th17	0.76% (0.18-1.54)	0.54% (0.25-0.98)	0.428
Th22	0.16% (0.09-0.36)	0.18% (0.06-0.76)	0.006 *

Cytokines and soluble molecules data were obtained from 35 MPM patients before and after RHRT, by multiplex and single ELISA assays. Immunophenotyping was performed on 27 MPM patients before and after RHRT, through flow cytometry. Gating plots used to identify the various immune cell populations were reported in **Supplementary Figure S2**. A significant difference was considered for *p<0.05, evaluated through Wilcoxon test for paired samples. By applying Bonferroni correction significance was considered for **p<0.002.

CTL, cytotoxic T lymphocytes; IFN, interferon; IL, interleukin; IQR, interquartile range; RHRT, radical hemithoracic radiation therapy; Th, T helper; TNF, tumor necrosis factor; Treg, regulatory T cells; w/o without.

### 3.3 Increased T cell responses against MPM-associated antigens after RHRT

**Figure 3:**
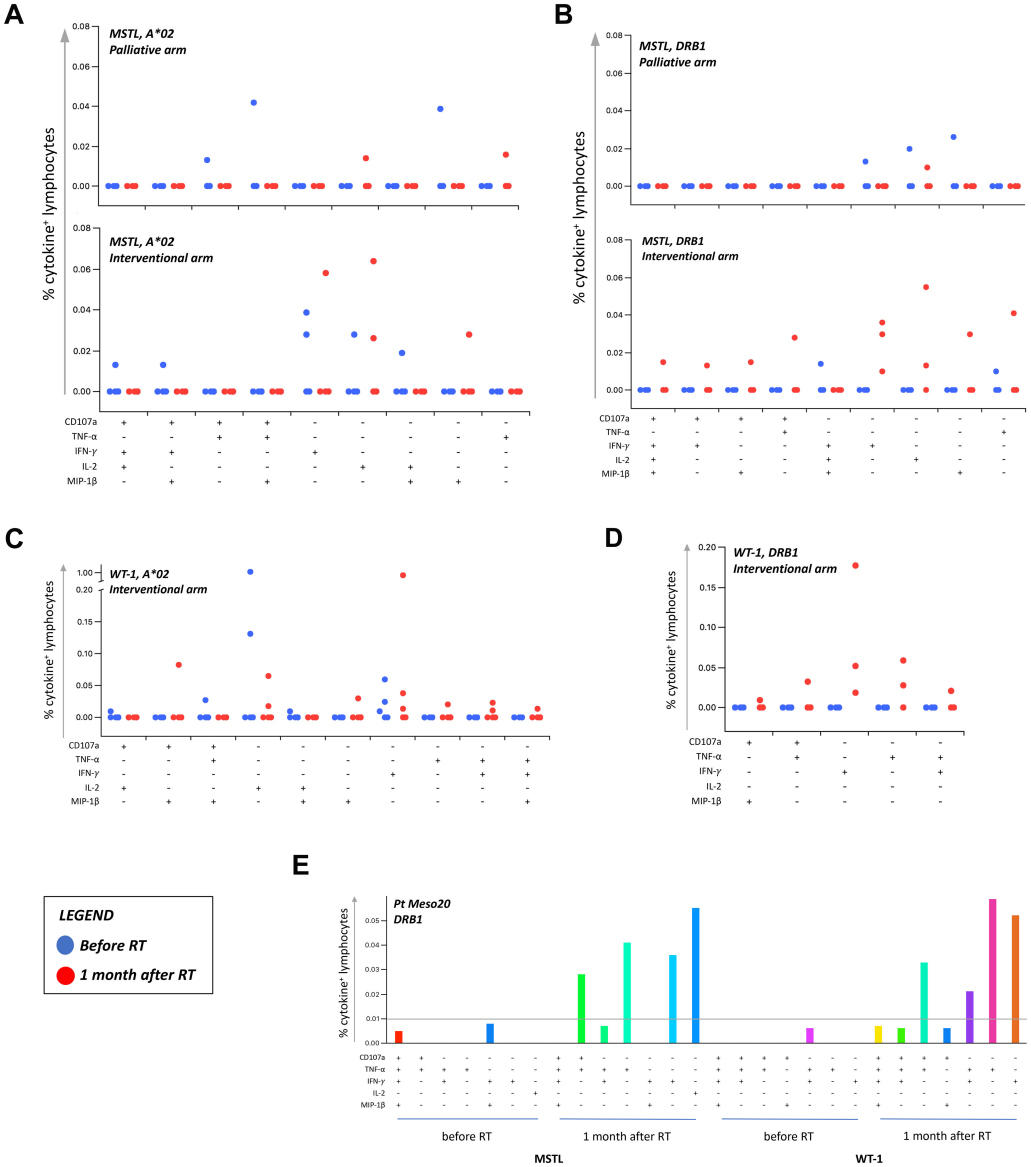
Tumor-specific T cell responses in MPM patients before and after RT. T cell responses against MPM-associated antigens (MSTL and WT1) were monitored through flow cytometry in MPM patients before and after (1 month) RT. The percentage of cytokine+ T cells was considered positive if at least doubled compared to the percentage of cytokine+ T cells after stimulation with a negative peptide control (HIV). Graphs in **(A–D)** reported the percentage of T-cells positive to at least one of the markers investigated (CD107a, TNF-α, IFN-γ, IL-2, and MIP-1β). A cut-off of 0.01% was set up to discriminate a positive population. Each dot represents the percentage of positive T-cells detected in each patient before (blue dots) and after (red dots) RT, following the stimulation with HLA-A*02 (A02)- or HLA-DRB (DRB)-restricted peptides. **(E)** Representative histograms of boolean analysis performed in one patient treated with RHRT and showing polyfunctional T cells after stimulation with HLA class-II restricted MSTL and WT1-derived peptides, before and after RHRT. Each bar represents a population of T cells producing a distinct set of cytokines or expressing CD107a as reported below. MSTL, mesothelin; RT, radiation therapy; WT-1, Wilms Tumor-1.

To evaluate whether the potential immunogenic modulation of high doses of RT could induce an anti-tumor “vaccine-like” effect, we monitored the presence and the amount of circulating CD4^+^ and CD8^+^ T cell responses against known epitopes derived from the MPM-associated antigens mesothelin and WT-1 before and 1 month after RT. These analyses were performed by flow cytometry in 43 MPM patients included in the randomized phase III trial described by Trovò et al. (35). **Supplementary Table S8** summarized the main clinical characteristics of these patients. Considering the available sample size for analysis, and based on the HLA background, we evaluated the presence of mesothelin-specific and WT-1-specific HLA class I-restricted CD8^+^ T cell responses in 18 and 17 patients, who carry the HLA-A*02 allele, respectively. CD4^+^ T cells specific for mesothelin were analyzed in 38 patients showing a DRB1 allele, and CD4^+^ T cells specific for WT-1 were monitored in 10 patients characterized by a DRB1*01 and/or a DRB1*04 allele (**Supplementary Table S3**), based on the selection of mesothelin and WT-1 DRB1-restricted epitopes already described in the literature (44, 45). The percentage of patients with a positive T cell response is summarized in **Table 3** and **Supplementary Table S9**. Interestingly, we noticed a significant increase in the number of patients exhibiting WT-1-specific CD4^+^ T cells after RT compared to the pre-treatment analysis, but only among patients included in the interventional arm receiving RHRT (p=0.046). As reported in **Table 3**, in a low but detectable number of patients (from 0 to 40%), we found a positive signal to at least one of the aforementioned antigens already before RT treatment, thus confirming the presence of spontaneous T-cell responses in MPM patients elicited by the tumor itself. Interestingly, in the interventional arm the number of antigen-specific T cells producing at least one of the tested cytokines appeared higher after RT (red dots) compared to the levels measured before treatment (blue dots; **Figures 3A–D**, interventional arm graphs). Moreover, mesothelin-specific T cells detected in patients treated with RHRT appeared more abundant compared to those detected in patient treated with palliative RT (**Figures 3A, B**). Following RHRT, MPM antigen-specific T cells displayed enhanced polyfunctional activity, producing a broader set of cytokines per cell compared to pre-RT levels (**Figure 3E**; example obtained from a single patient treated with RHRT). These results suggested that radical doses of RT may have a stronger impact on the stimulation of anti-tumor T-cell responses compared to palliative doses of radiation. However, focusing the analysis solely on 2 single TAA, mesothelin and WT-1, did not provide a comprehensive view of T-cell specificities necessary to monitor the anti-tumor T-cell response.

**Table 3:** T cell responses specific for MPM-associated antigens in MPM patients before and after RT.

Therapy arm	T cell specificity	Positive response (patients tested) *Percentage*		Chi-squared test
		Before RT	1 month after RT	
**Palliative arm**	anti-MSTL CD8^+^	2 (8) *25%*	3 (8) *37.5%*	0.590
	anti-MSTL CD4^+^	2 (19) *10.5%*	1 (19) *5.3%*	0.547
	anti-WT1 CD8^+^	0 (7) *0%*	0 (7) *0%*	1.000
	anti-WT1 CD4^+^	0 (4) *0%*	0 (4) *0%*	1.000
**Interventional arm**	anti-MSTL CD8^+^	3 (10) *30%*	3 (10) *30%*	1.000
	anti-MSTL CD4^+^	2 (19) *10.5%*	4 (19) *21%*	0.373
	anti-WT1 CD8^+^	4 (10) *40%*	5 (10) *50%*	0.653
	anti-WT1 CD4^+^	0 (6) *0%*	3 (6) *50%*	0.046 *

MSTL, mesothelin; RT, radiation therapy; WT-1, Wilms Tumor-1.

*p<0.05.

### 3.4 RHRT influence on the clonality of the TCR repertoire

**Figure 4:**
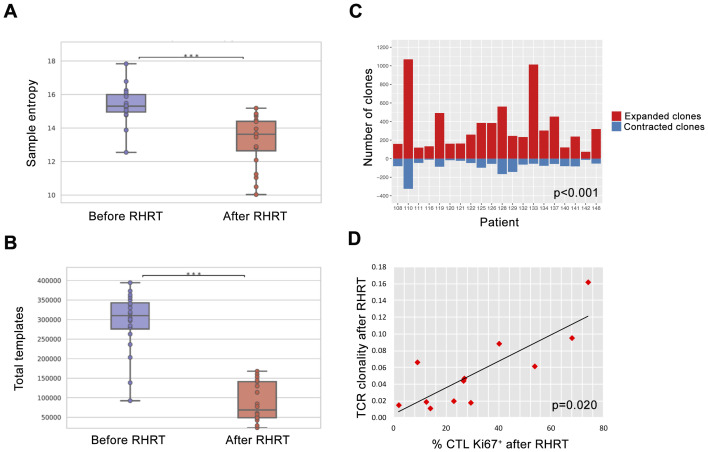
TCR repertoire analysis before and after RHRT. **(A)** Evaluation of the sample entropy for the TCR repertoire measured before and after RHRT in 20 patients. For each box plot, points represent the value of a single sample. Lines represent median values, while boxes show the IQR and the whiskers the lowest and the highest values. ***, p<0.001 **(B)** Number of total templates obtained in each patient before and after RHRT. **(C)** Number of T-cell clones significantly expanded (red) or contracted (blue) when comparing TCR repertoire obtained before and after RHRT in each patient. **(D)** Correlation analysis between the value of TCR clonality and the proportion of CTLs (CD8^+^ T cells) expressing the proliferation marker Ki-67, both measured after RHRT. CTL, Cytotoxic T Cells; RHRT, Radical Hemithoracic RadioTherapy; TCR, T-cell Receptor.

In order to better define the impact of radical radiation doses on the global T-cell response, we characterized the whole TCR repertoire in MPM patients before and after RHRT. This analysis allowed us to determine for each sample the number of T-cell clones belonging to the repertoire at a single time-point (sample diversity), and the extent of expansion of all specific T-cell clones in the repertoire (sample clonality). Twenty out of 35 patients enrolled in the MESORTIBO study and treated between 2020 and 2022 were characterized by TCRB Next Generation Sequencing (NGS) analysis before and at the end of RHRT. Globally, 6,117,075 productive templates were obtained from a total of 40 blood samples. The average count of total rearrangements/sample was 105,932 ± 77,113 (range 10,453-296,803), while productive TCR rearrangements per sample were 85,592 ± 62,681 (range 8,783-246,998). No correlation was noted with age, sex, tumor histology, nor tumor site (left or right) for any of the following parameter: sample Simpson clonality (**Supplementary Table S10**), total templates, maximal frequency, nor sample diversity index as entropy (data not shown), calculated before and after RHRT. Paired samples obtained from the same patient at different time points showed an average Morisita similarity index of 0.57 ± 0.28. Conversely, comparing samples from different patients we detected an average Morisita similarity index of 0.00002 ± 0.0003, significantly lower than the paired samples Morisita index (p<0.001). The Simpson clonality index accounted for a mean value of 0.055 ± 0.035 and 0.063 ± 0.044 before RHRT and after RHRT, respectively (p=0.400). Samples obtained before RHRT showed a significantly higher entropy, compared to samples collected at the end of RHRT, probably because of the higher number of total templates obtained before RHRT (**Figures 4A, B**; p<0.001). To overcome the potential bias of the different templates number between samples obtained before RHRT and those obtained after RHRT, we performed downsampling by the ImmunoSEQ Analyzer tool, i.e. each sample repertoire was downsampled to a common number of templates. A DownSampled (DS) Productive Simpson Clonality was calculated with this tool and used from this point forward to further analyses. Next, we wanted to evaluate whether the effect of RHRT could influence the global TCR repertoire inducing a reshuffle of TCR characteristics and specificities or just favored the expansion of specific T-cell clones. We first characterized the Complementarity-Determining Regions (CDR3) length and the usage of TRBV (TCR-β variant) and TRBJ (TCR- β joining) genes in TCR sequences obtained before and after RHRT in the global cohort of patients. We noticed a comparable distribution of both CDR3 lengths and TRBV and TRBJ genes families between the two time-points (**Supplementary Figure S4**), thus suggesting that RHRT did not revolutionize the TCR repertoire, but probably altered the frequency of selected T-cell clones. Therefore, we proceeded by comparing the abundance of the same T-cell clones detected in paired samples (before and after RHRT in the same patient). We noticed an increased number of significantly expanded clones after RHRT (344 ± 274) compared to the number of significantly contracted clones (79 ± 70; p=0.0001; **Figure 4C** and **Supplementary Figure S5**), thus suggesting a potential boost induced by therapy in the proliferation of selected T-cell clonal populations. Interestingly, the values of TCR clonality calculated after RHRT showed a strong positive association with the percentage of CTLs, and an inverse relationship with the percentage of Th cells (**Table 4**). Moreover, TCR clonality after RHRT positively correlated with the CTL/Treg ratio, with the percentage of proliferating T cells, and in particular with the proportion of proliferating CTLs (**Table 4** and **Figure 4D**). In contrast, the Simpson Clonality Index measured before RHRT showed a negative correlation solely with the percentage of Th1 cells producing TNF-α (Spearman rho -0.569, p=0.040; data not shown). Previous evidence reported a positive correlation between TCR clonality and HED, the sequence divergence of the HLA-I genotype, as greater divergence enables the presentation of a more variable array of immunopeptides (40). However, in our population, we did not observe any correlation between TCR clonality measured both before and after RHRT and HLA background calculated as HED for HLA-A and B alleles and as median HED (before RHRT Spearman Rho = 0.329, p value = 0.156; after RHRT Spearman Rho = -0.178, p value = 0.466; **Supplementary Table S4**). Moreover, neither TCR clonality nor HED were associated with OS (**Supplementary Table S11**). To investigate the specificity of expanded T cell clones, which *bona fide* mainly affected TCR clonality measured after RHRT, we performed several bioinformatics analyses comparing the TCR sequences obtained in MPM patients with the following datasets: (i) a large-scale database of TCRβ sequences from natural and synthetic exposure to SARS-CoV-2 (46); (ii) TCRβ sequences obtained from healthy donors (38); (iii) TCRβ sequences characterized in a different cohort of mesothelioma patients undergoing immunotherapy (47). Given that patients’ samples were collected between July 2020 and August 2022, during the course of the COVID19 pandemic and the SARS-CoV-2 vaccination campaign, both of which induce an expansion of TCR clones specific for SARS-CoV-2-derived antigens, we used the COVID search tool (ImmunoSEQ Analyzer) to identify all SARS-CoV-2-associated TCR sequences in post-RHRT samples. This step allowed us to exclude their main contribution to the TCR clonality measured at this time point. The median global frequency of all identified SARS-CoV-2-associated templates (median number=196, [IQR, 59-547]) was 0.0067 (IQR, 0.0042-0.0137), significantly lower compared to the sum of the frequencies of the 20 most abundant TCR templates identified after RHRT (median, 0.0996 [IQR, 0.0197-0.417]; p < 0.001; **Supplementary Table S12**). This finding led us to speculate that the Simpson clonality indexes calculated after RHRT in our cohort were not significantly influenced by SARS-CoV-2-infection and/or vaccination. To exclude the possibility that these most abundant templates were associated with a generic infection commonly present in the general population, we searched for them within the TCR repertoire of 119 healthy donors as detailed in the methods section. Only 25 sequences (6.26%) were common to both groups, and each sequence was detected in a median of 4 healthy donors (range 1–97) at a low median frequency (median, 7; range, 5-26 templates). Finally, to investigate whether the TCR sequences identified in our patients may be suggestive of a MPM-specific anti-tumor immune response, we compared our data with those obtained by Desai et al, who described the TCR repertoire dynamics in MPM patients treated with immunotherapy and its association with survival (47). Their analysis suggested that the expansion of TCR repertoire following treatment with ICI could be associated with improved survival. Specifically, they found two TCR clusters significantly associated with survival, though without identifying a unique antigen-specificity (47). We thus compared our samples with those analyzed by Desai et al, focusing on TCR sequences from MPM patients before and after ICI treatment, using the ImmunoSEQ Analyzer. Applying the sample overlap tool, we selected samples combinations with a Morisita similarity index higher than 0.0001, corresponding to the 95^th^ percentile of all the Morisita indexes calculated when comparing the two patients cohorts (MESORTIBO and the cohort described by Desai et al.). These comparisons included samples obtained before or after RHRT (our cohort) and samples collected before or after ICI therapy (Desai et al. cohort). **Supplementary Table 13** reported the number of common entries found for each combination of 3 samples: one from the MESORTIBO patients (before or after RHRT), and paired samples from the same patient (before and after ICI) in Desai et al.’s cohort. We then selected TCR sequences that increased by at least 1.5-fold after ICI therapy and searched their antigen specificity looking for the presence of the exact (100% identity) CDR3 aminoacid relative sequences in 3 databases (VDJdb (https://vdjdb.cdr3.net/search) (48), McPAS-TCR (https://friedmanlab.weizmann.ac.il/McPAS-TCR/) (49), and TCR Match (http://tools.iedb.org/tcrmatch/) (50)). **Supplementary Table S13** provides the number of sequences with at least one known epitope-specificity and those that remain uncharacterized. Overall, we obtained 1,545 unique TCR sequences with unknown specificity, which were subsequently compared with the TCR sequences from healthy donors, as described in the materials and methods section. We found 649 (42%) common sequences, present in a median of 5 donors (range 5-12) with a median frequency of 6 (range 5-29). Excluding them from the unknown sequences described in **Supplementary Table 13**, we obtained 896 unknown unique sequences detected among MPM patients but not in healthy donors. Further analysis of sequences characterized in TCR databases and shared among MPM patients, revealed a range of viral specificities (e.g. EBV, CMV, Flu, SARS-CoV-2, Hepatitis B virus [HBV]; data not shown). Notably, we also detected several TCR sequences specific for TAA and other antigens already described in tumor tissue, as reported in **Table 5**. Intriguingly, among the identified TAA recognized by TCR sequences shared between our MPM patients and those included in the cohort of Desai et al, we observed the MPM-associated antigen WT-1, which has previously been able to induce functional anti-tumor T cell responses after RHRT in our earlier cohort of patients (**Figure 3D**).

**Table 4:** Correlation between Simpson Clonality Index and immune parameters, both detected after RHRT.

Parameter after RHRT	Spearman rho with TCR clonality (p value)
T cells (CD3)	0.070 (0.834)
Th cells (CD4)	-0.804 (0.002) *
CTLs (CD8)	0.909 (<0.001) *
Tregs	-0.168 (0.604)
CTL/Treg	0.622 (0.030) *
Th1 IFN-γ	-0.161 (0.619)
Th1 TNF-α	-0.448 (0.147)
Th17	-0.273 (0.391)
Th22	-0.287 (0.366)
T cells PD-1^+^	0.039 (0.905)
Th cells PD-1^+^	-0.112 (0.733)
CTL PD-1^+^	0.420 (0.177)
T cells Ki67^+^	0.713 (0.010) *
Th cells Ki67^+^	0.420 (0.177)
CTL Ki67^+^	0.685 (0.017) *

A significant (*) difference was considered for p<0.05, evaluated through Spearman rho correlation analysis.

CTL, cytotoxic T lymphocytes; IFN, interferon; TNF, tumor necrosis factor; RHRT, radical hemithoracic radiation therapy; TCR, T-Cell Receptor; Th, T helper; Treg, regulatory T cells.

**Table 5:** Tumor-Associated Antigens specificities identified for common TCR sequences found in both MPM patients of the MESORTIBO cohort and MPM patients described in Desai et al (47).

Tumor-Associated Antigens TCRs specificities	
AKAP13	NY-ESO-1
APBB2	OR14C36
ATP6AP1	p53
BST2	PABPC1
CDK4	PGM5
FNDC3B	PLA2G6
GALC	PLXNB1
GANAB	PMEL (gp100)
GNL3L	SEC24A
IGF2BP2	SF3B1
INTS1	SMARCD3
KAT6A	SREBF1
LCP1	ST6GALNAC2
MAGEA6	TKT
Melan-A/MART-1	TRPV3
NRAS	TRPV4
NSDHL	WT-1

AKAP13, A-Kinase Anchoring Protein 13; APBB2, Amyloid Beta Precursor Protein Binding Family B Member 2; ATP6AP1, ATPase H+ Transporting Accessory Protein 1; BST2, Bone Marrow Stromal Cell Antigen 2; CDK4, Cyclin-dependent kinase 4; FNDC3B, Fibronectin Type III Domain Containing 3B; GALC, Galactosylceramidase; GANAB, Glucosidase II Alpha Subunit; GNL3L, G protein nucleolar 3 like; gp100, glycoprotein 100; IGF2BP2, Insulin Like Growth Factor 2 MRNA Binding Protein 2; INTS1, Integrator Complex Subunit 1; KAT6A, K(lysine) acetyltransferase 6A; LCP1, Lymphocyte Cytosolic Protein 1; MAGEA6, Melanoma-Associated Antigen 6; MART1, Melanoma Antigen Recognized by T cells 1; NRAS, neuroblastoma ras viral oncogene homolog; NSDHL, NAD(P) dependent steroid dehydrogenase-like; NY-ESO1, New York esophageal squamous cell carcinoma 1; OR14C36, Olfactory Receptor Family 14 Subfamily C Member 36; PABPC1, Poly(A) Binding Protein Cytoplasmic 1; PGM5, Phosphoglucomutase-like protein 5; PLA2G6, Phospholipase A2 Group VI; PLXNB1, Plexin B1; PMEL, premelanosome protein; SEC24A, SEC24 homolog A; SF3B1, splicing factor 3b subunit 1; SMARCD3, SWI/SNF Related, Matrix Associated, Actin Dependent Regulator Of Chromatin, Subfamily D, Member 3; SREBF1, Sterol Regulatory Element Binding Transcription Factor 1; ST6GALNAC2, ST6 N-Acetylgalactosaminide Alpha-2,6-Sialyltransferase 2; TCR, T-Cell Receptor; TKT, Transketolase; TRPV, Transient Receptor Potential Cation Channel Subfamily V Member; WT-1, Wilms Tumor 1.

## 4 Discussion

Recently, some authors suggested a synergistic role of IT and RT, in several settings of oncologic diseases (51). This is mainly due to the potential immunogenic role of RT, which may favor the release of TAA and neoantigens, together with Damage-Associated Molecular Patterns (DAMPs), thus lastly stimulating the activation and proliferation of anti-tumor T cells (52). Since this effect may depend on RT dose and fractionation (53), it would be helpful to find a strategy able to identify circulating biomarkers of RT-induced anti-tumor immunity. Therefore we evaluated several immunomonitoring methods in a population of MPM patients undergoing RHRT and mainly affected by a malignancy of an epithelioid histotype (**Supplementary Figure S1**), which had showed a reduced benefit from immunotherapy compared to the sarcomatoid type (14). Moreover, in MPM available data demonstrated the possibility to integrate the RHRT with IT, and the feasibility of a combined treatment between curative RT and IT has only been recently investigated (ClinicalTrials.gov ID: NCT02959463). In the present study, we observed both a contribution of lymphocytes in the prognosis after RT treatment, and a modulation of the T cell phenotype and function after RT, thus suggesting a major role of T lymphocytes in the mechanism of action of RHRT in MPM patients.

We first investigated three systemic immune-inflammatory biomarkers, LMR, NLR, and SII, obtained by blood count data analysis. The LMR serves as a valuable hematological parameter that offers a window into the delicate balance between lymphocytes and monocytes, two essential types of white blood cells. An elevated LMR may indicate a robust immune response, whereas a decreased LMR could suggest immune dysfunction. By evaluating the LMR before and after RT, we aimed to uncover the treatment’s effects on the immune system and their potential consequences for patient outcomes. Furthermore, the NLR and SII were calculated to provide additional context regarding inflammation and systemic stress. These metrics, in conjunction with the LMR, offer a more comprehensive understanding of the immune setting and its response to RT. By analyzing these ratios and indices, we strive to enhance our knowledge of the intricate interplay between the immune system and radiotherapeutic interventions. This knowledge has the potential to promote the development of more personalized and targeted treatment strategies in the future, ultimately improving patient outcomes and tailoring therapies to individual needs. We noticed that LMR ratio calculated before RHRT was associated with OS, indeed patients characterized by a higher LMR showed a better outcome compared to those having a lower ratio (**Figure 1**), while NLR and SII did not demonstrate any prognostic significance. Low LMR was reported as an independent marker of poor prognosis also in other MPM cohorts, showing a superior prognostic ability compared to other inflammation-based prognostic scores (54, 55). Due to the multimodal treatment employed in the management of mesothelioma patients, the prognostic role ascribed to LMR in these studies may be associated also to chemotherapy and/or surgery approaches. Interestingly, high pre-treatment LMR levels were associated with longer OS in other thoracic diseases treated only with thoracic radiotherapy (56, 57). LMR values strongly depends positively on the number of total lymphocytes, and negatively on the number of total monocytes, and we previously reported that the variation in T lymphocytes after RHRT was positively associated with OS in MPM patients (33). Thus we hypothesized that lymphocytes rather than monocytes may have a role in the response to radiation, and we focus our attention on serum molecules and circulating cells involved in the anti-tumor immune response, which were significantly modified after RHRT compared to palliative RT (33).

In the present study we confirmed an increase in serum IL-10 levels at the end of treatment (**Table 2**) as already reported in our previous analysis (33). IL-10 was initially considered as a marker of immune suppression, for its ability to inhibit cytokine secretion, antigen presentation and CD4^+^ T cell activation (58). IL-10 is mainly produced by monocytes, mast cells, Th2 cells, and Treg (59). Interestingly, after RHRT we noticed also a significant increase in Treg, which was associated with a limited success of irradiation (15Gy) in a mouse model of mesothelioma (60). We had already observed this Treg boost in our previous analysis, that, however, was evident also in the cohort of patients treated with palliative RT (33), thus suggesting that this effect is independent of radiation dose and is probably just a consequence of irradiation (27, 52). This could be due to the fact that Treg showed a higher radioresistance compared to other immune cells (61). Their increase after radiotherapy was reported in mouse cancer models treated with irradiation, as well as in other cohorts of cancer patients undergoing radiotherapy (32). These data are consistent with the known homeostatic immunosuppressive mechanism triggered by RT, that is counterbalanced by its immunogenic effect (62). Interestingly, it was demonstrated that the removal of Treg can shift the balance in favor of radiation-induced anti-tumor immunogenic effects (60, 63). Beyond the immunosuppressive activity ascribed to IL-10, Mumm and colleagues demonstrated its ability to induce mechanisms involved in anti-tumor immune surveillance, in particular by favoring the expression of IFN-γ by Th1 cells and activating anti-tumor CD8^+^ T cells (64). The pleiotropic role of IL-10 seems to depend on the context and the concentration, but it is currently believed that this cytokine can promote the activation of tumor-resident CD8^+^ T cells (65). In our analysis, the same trend was observed also for serum IFN-γ and sPD-L1, which showed a more prominent boost after RHRT compared to IL-10 (**Table 2**). A contemporary increase of both serum IL-10 and IFN-γ was observed also by Gkika et al. in patients affected by thoracic malignancies and treated with radiation therapy (66). The enhancement of IFN-γ may be considered a marker of CTL activation as a consequence of radiation-induced T-cell tumor infiltration and activation (67). IFN-γ in turn, induces the upregulation of PD-L1 expression on tumor cells, a mechanism proposed as responsible for acquired resistance to fractionated RT (67). Consistently, we also noticed an increase in the soluble levels of PD-L1 after RHRT, speculating that this molecule could derive from tumor cells and/or other cells present in the tumor microenvironment that are perturbed by radiation. Intriguingly, an *in vitro* study evaluating the expression of PD-L1 on mesothelioma cell lines showed an increased surface expression of this molecule 72 hours after cell irradiation (8 Gy) (68). At the same time, irradiation was also able to increase the PD-1 expression on CD8^+^ T cells infiltrating the tumor in mouse models of different cancer types and *ex vivo* in TILs from human carcinomas (69). In our cohort, the percentage of PD-1^+^ CD8^+^ circulating T cells together with the amount of PD-1^+^ CD4^+^ T cells and globally PD-1^+^ CD3^+^ T cells, significantly improved at the end of RHRT and even more 1 month after treatment (**Figure 2**). Intriguingly, several papers identified neoantigen-specific T cells preferentially enriched in the proportion of CD8^+^ and CD4^+^ T cells expressing PD-1, even suggesting the monitoring of PD-1^+^ CD8^+^ T cells as a non-invasive surrogate of neoantigen-reactive T cells residing within the tumor (70–72). We thus speculated that RHRT may induce the release of neoantigens by tumor cells that in turn stimulated the priming and expansion of neoantigen-specific T cells, represented by the boosted PD-1^+^ CD8^+^ and CD4^+^ T cells observed in the periphery. Consistently, when comparing the TCR repertoire of paired samples, after RHRT we noted several significantly expanded T cell clones, which were absent in samples obtained before RT (**Supplementary Figure S5**, red dots on the Y axis).

Globally, after RHRT we observed a significantly higher number of expanded T cell clones compared to the amount of contracted ones (**Figure 4C**), including also TCR sequences already observed before therapy. Currently, due to the reduced number of studies on TAA, known TCR specificities are mainly linked to viral or bacterial infection. Moreover, it is known that a large fraction of T-cell clones that determine the TCR repertoire of a cancer patient is not related to the anti-tumor immune response (73). Even if we cannot confirm the tumor specificity of TCR sequences, which significantly expanded after RHRT, we noticed that only a minority (6.26%) of them could be found in a control group of healthy donors. Moreover, the frequency of SARS-CoV-2-specific sequences did not have a significant impact on the final clonality measured after RHRT (**Supplementary Table S12**), despite the enrollment time overlapped the COVID19 pandemic and anti-SARS-CoV-2 vaccination period. We thus speculated a tumor specificity of at least some of these sequences, that may imply the presence of spontaneous T-cell responses elicited by the tumor itself, and boosted by RHRT. These data are in line with the results obtained in the cohort of MPM patients included in the randomized trial, where we documented the presence of functional CD8^+^ and CD4^+^ T-cell responses against mesothelin and WT-1, both TAA expressed by mesothelioma (74–76), already before treatment in a proportion of MPM patients (**Figure 3**). Interestingly, RHRT, but not palliative RT, seemed to have both the ability to increase the number of patients showing a TAA-specific T cell response, and to improve the quantity and quality of these responses by favoring the induction of a polyfunctional T cell activity (**Figure 3**). In viral infection and vaccination procedures multifunctional CD8^+^ and CD4^+^ T cells were considered optimized for effector function and associated with enhanced protection (77), thus supporting the improved efficacy of the observed polyfunctional anti-tumor T cells. We previously reported that high doses of RT were able to induce polyfunctional TAA-specific T-cell responses in metastatic breast cancer patients (78), who experienced a long-term progression-free survival after radical RT (79). Consistently, other clinical studies demonstrated the ability of high doses of RT to induce anti-tumor CD8^+^ T cells producing IFN-γ and other pro-inflammatory cytokines in the periphery (80–82), while preclinical studies reported the increase in CD8^+^ TILs producing cytokine and cytolytic enzymes after RT (83–86). Notably, in our study, TCR clonality after RHRT was positively associated with the percentage of CD8^+^ T cells, particularly with those expressing the proliferation marker Ki67 (**Table 4**). The significant enhancement of CD8^+^Ki67^+^ T cells after RHRT supports the main contribution of CTLs cells in the definition of the TCR repertoire after therapy. Similarly, Gkika et al. reported a significant increase in CD8^+^Ki67^+^ and CD4^+^Ki67^+^ T cells in NSCLC patients treated with SBRT (82). They associated these variations with a global RT-induced lymphopenia. We also noticed a significant reduction of total T cells after RHRT (**Table 2**). Interestingly, the lymphopenia induced by RT was previously correlated with the increased T-cell proliferation, which likely includes tumor-specific T-cells (71, 72, 82, 87, 88).

We could not definitively confirm that the increased percentage of CD8^+^Ki67^+^ T cells observed after RHRT, and associated with TCR clonality was mainly enriched in anti-tumor T cells. However, the comparison of our TCR repertoire with those of other MPM patients undergoing immunotherapy (47) and a cohort of healthy donors (38), revealed a high prevalence of common TCR sequences among MPM patients belonging to different cohorts, partially already described in the literature (**Supplementary Table S13** and **Table 5**) and largely still of unknown specificity, but likely linked to tumor antigens due to their absence in healthy people. Beyond viral and bacterial antigens, we interestingly found several TAA (**Table 5**) associated with TCR sequences both present in our MPM patients and described in the cohort of Desai et al. among TCR clones increased after ICI therapy. Intriguingly, CDK4 (89), NRAS (90), NY-ESO-1 (91), p53 (92), SF3B1 (93, 94), and WT-1 (74) antigens had been previously described in association with mesothelioma, thus suggesting that this tumor may be able to induce T cell responses against these TAA. Notably, T-cell responses against NY-ESO-1 and WT-1 were documented in MPM patients (91, 95), and in our cohort of patients we reported improved WT-1 specific CD4^+^ T cells producing IFN-γ or TNF-α after RHRT (**Figure 3D**). Several clinical trials employing peptide- or dendritic cell-based cancer vaccines targeting WT-1 are currently ongoing for the treatment of mesothelioma alone or in combination with immunotherapy (ClinicalTrials.gov ID: NCT04040231, NCT02649829, NCT05765084) (96, 97). In this respect, the possible vaccine-like effect induced by RHRT in MPM patients included in the present study, may suggest a synergistic effect of this kind of RT with ICI therapy, which could exploit the immunogenic consequence of irradiation.

Our results could not be considered definitive because our study has several limitations. Indeed, the analysis of functional T-cell responses was performed only against 2 TAA, thus strongly restricting the characterization of anti-tumor immunity. Moreover, TCR repertoire was investigated only at the end of the radiotherapeutic treatment, while probably it can still change 1 month after RT, as the reshuffle of the TCR may require time (98). Furthermore, we performed the whole immunomonitoring in the periphery, without investigating the tumor microenvironment due to the complex access to the pleura. However, it was demonstrated that the TCR repertoire in the periphery could *bona fide* mimic the TCR within the tumor (99).

In conclusion, in a cohort of patients affected by an epithelioid or biphasic MPM, our results suggested that RHRT could be able to induce the expansion of CD8^+^ T cell clones, with at least a fraction of them showing an anti-tumor specificity. These findings indicate a potential synergistic effect of this type of RT with immunotherapy, currently employed for sarcomatoid MPM. In addition, anti-CTLA4 and anti-PD-1 ICI could counteract the immunosuppressive effects induced by RHRT, thus favoring the final effective activation of the anti-tumor immune response. Finally, to reduce the risk of a cumulative RT- and immunotherapy-induced toxicity, the RHRT could be combined with a monovalent bispecific PD-1/CTLA4 antibody (100) currently under evaluation for the treatment of MPM (ClinicalTrials.gov ID: NCT06097728). We may still exploit the synergy between the two treatment modalities, reducing the risk of lung fibrosis, due to its lower toxicity profile.

## Data Availability

The data presented in the study are deposited in the immuneACCESS repository, accession number DOI: 10.21417/AR2025S at http://clients.adaptivebiotech.com/pub/revelant-2025-s and in the Figshare repository at: https://figshare.com/articles/dataset/_b_Radical_hemithorax_radiotherapy_induces_an_increase_in_circulating_PD1_b_sup_strong_strong_sup_b_T_lymphocytes_and_in_the_soluble_levels_of_PD-L1_in_Malignant_Pleural_Mesothelioma_patients_a_possible_synergy_with_PD-1_PDL1_targeting_tr/28678397.
