# Supplementary material for: Radical hemithorax radiotherapy induces an increase in circulating PD-1+ T lymphocytes and in the soluble levels of PD-L1 in malignant pleural mesothelioma patients: a possible synergy with PD-1/PD-L1 targeting treatment?
Source: Front Immunol. 2025 Apr 1;16:1534766. doi: 10.3389/fimmu.2025.1534766 (PMC11997449; doi:10.3389/fimmu.2025.1534766)
Supplement: Supplementary file 1 [file DataSheet1.pdf]

Radical hemithorax radiotherapy induces an increase in circulating PD-1$^+$ T lymphocytes and in the soluble levels of PD-L1 in Malignant Pleural Mesothelioma patients: a possible synergy with PD-1/PD-L1 targeting treatment?

Revelant A[1], Gessoni F[1*], Montico M[2], Dhibi R[3], Brisotto G[3] , Casarotto M[3], Zanchetta M[2], Paduano V[4], Sperti F[4], Evangelista C[4], Giordari F[3], De Re V[3], Trovo' M[5], Minatel E[1], Mascarin M[1], Steffan A[3], Muraro E[3]

Supplementary Material

Supplementary Tables and Figures

Tables

Supplemental Table 1. List of epitopes derived from MPM-associated antigens or viral antigens as controls

MPM-associated antigens	HLA allele	Epitope aminoacid position	Epitope sequence	References
Mesothelin	-A*0201	Mesothelin 20-28	SLLFLLFSL	(1)
	-DRB1	Mesothelin 531-545	FMKLRTDAVLPLTVA	(2)
WT-1	-A*0201	WT-1 126-134	RMFPNAPYL	(3)
	-DRB1*0101<br>-DRB1*0401	WT-1 124-138	PQQMGSDVRDLNALL	(4)
**Negative controls**				
HIV	-A*0201	Gag p17 77-85	SLYNTVATL	
	-DRB1	p24 164-181	YVDRFYKTLRAEQASQEV	
**Positive controls**				
Flu	-A*0201	Matrix Protein 1 58-66	GILGFVFTL	
EBV	-A*0201	BMLF1 280-288	GLCTLVAML	
	-DRB1	EBNA1 514-539	KTSLYNLRRGTALAIPQCRLTPLSRL	

Abbreviations: EBV, Epstein-Barr Virus; Flu, Influenza; HIV, Human Immunodeficiency Virus; HLA, Human Leukocyte Antigen; MPM, Malignant Pleural Mesothelioma; WT-1, Wilms Tumor-1.

Supplemental Table 2. Antibodies for flow cytometry

Antigen	Fluorochrome	Isotype	Clone	Brand
**CD3**	FITC	Mouse IgG1, κ	UCHT1	BD
**CD4**	APC-Cy7	Mouse IgG1, κ	RPA-T4	BD
**CD8**	BV421	Mouse IgG1, κ	RPA-T8	BD
**CD25 (IL-2Rα)**	APC	Mouse IgG1, κ	M-A251	BD
**CD45RA**	Alexa Fluor 700	Mouse IgG2b, κ	HI100	BD
**CD107a**	FITC	Mouse IgG1 κ	H4A3	BD
**CD279 (PD-1)**	PE-CF594	Mouse IgG1, κ	EH12.1	BD
**IFN-γ**	PE-Cy7	Mouse IgG1, κ	4S.B3	BD
**TNF-α**	PE	Mouse IgG1, κ	MAb11	BD
**MIP-1β**	PE and Alexa Fluor 700	Mouse IgG1, κ	D21-1351	BD
**IL-2**	APC	Rat IgG2a, κ	MQ1-17H12	BD
**IL-17A**	Alexa Fluor 647	Mouse IgG1, κ	N49-653	BD
**IL-22**	PerCP-eFluor ®710	Mouse IgG1, κ	22URTI	Affymetrix eBioscience
**Foxp3**	PE	Mouse IgG1, κ	236A/E7	Affymetrix eBioscience
**Ki-67**	PE-Cy™7	Mouse IgG1 κ	B56	BD

Abbreviations: APC, Allophycocyanin; BV, Brillant Violet; FITC, Fluorescein isothiocyanate; IFN, Interferon; IL, Interleuchin; PD, Programmed Death; MIP, Macrophage inflammatory protein; PE, Phycoerythrin; TNF, Tumor Necrosis Factor.

Supplemental Table 3. Characterization of HLA-class I and class II, and antigen tested for each patient included in the randomized trial.

Patient number	HLA-class I	Antigens tested for HLA class-I restricted T cell responses	HLA-class II	Antigens tested for HLA class-II restricted T cell responses	Arm
1			DRB1*03	MSTL	Palliative
4	A*02	MSTL, WT-1	DRB1*03	MSTL	Palliative
5	A*02	MSTL, WT-1	DRB1*03		Interventional
6			DRB1*03	MSTL	Palliative
7	A*02	MSTL, WT-1	DRB1*03	MSTL	Interventional
8			DRB1*01	WT-1	Palliative
13			DRB1*03	MSTL	Palliative
15			DRB1*04	MSTL, WT-1	Interventional
17			DRB1*01 DRB1*03	MSTL	Interventional
18			DRB1*04	MSTL, WT-1	Palliative
19	A*02	MSTL WT-1	DRB1*03	MSTL	Interventional
20			DRB1*04	MSTL, WT-1	Interventional
22			DRB1*01 DRB1*03	MSTL WT-1	Interventional
23	A*02	MSTL, WT-1	DRB1*03	MSTL	Interventional
26	A*02	MSTL WT-1	DRB1*03	MSTL	Palliative
29	A*02	MSTL, WT-1	DRB1*01, DRB1*03	MSTL, WT-1	Interventional
30	A*02	MSTL WT-1	DRB1*03	MSTL	Palliative
31			DRB1*03	MSTL	Interventional
32			DRB1*03	MSTL	Interventional
34	A*02	MSTL, WT-1	DRB1*03	MSTL	Palliative
35			DRB1*1501, DRB1*03	MSTL	Palliative
36			DRB1*03, DRB1*04	MSTL, WT-1	Palliative
37	A*02	MSTL, WT-1	DRB1*04	MSTL	Interventional
38	A*02	MSTL, WT-1	DRB1*03	MSTL	Palliative
39			DRB1*04	MSTL, WT-1	Interventional
43	A*02	MSTL, WT-1	DRB1*03	MSTL	Interventional
47			DRB1*1501, DRB1*03	MSTL	Palliative
49	A*02	MSTL, WT-1	DRB1*03	MSTL	Palliative
52			DRB1*03	MSTL	Interventional
53	A*02	MSTL WT-1	DRB1*03	MSTL	Interventional
55			DRB1*03	MSTL	Interventional
57	A*02	MSTL WT-1	DRB1*1501 DRB1*04		Interventional
59	A*02	MSTL, WT-1	DRB1*03	MSTL	Interventional
61			DRB1*03	MSTL	Palliative
62			DRB1*1501	MSTL	Interventional
63			DRB1*01	MSTL WT-1	Palliative
64			DRB1*1501	MSTL	Palliative
65	A*02	MSTL	DRB1*01; DRB1*03		Palliative
67			DRB1*03	MSTL	Palliative
73			DRB1*1501	MSTL	Palliative
76			DRB1*1501, DRB1*03	MSTL	Palliative
77			DRB1*01	MSTL, WT-1	Interventional
78	A*02	MSTL, WT-1			Palliative

Abbreviations: MSTL, mesothelin; WT-1, Wilms Tumor-1.

Supplemental Table 4. HLA-A and –B, and relative HED characterization in the MESORTIBO cohort.

Patient number	HLA-A*			HLA-B*			Mean HED
	1st allele	2nd allele	HED HLA-A	1st allele	2nd allele	HED HLA-B	
108	0301	2402	10.70	1801	3501	4.71	7.70
110	0101	1101	3.20	2705	3501	11.01	7.10
111	0201	6801	5.20	1801	5101	8.73	6.97
113	0201	2501	12.49	35150	44269	8.10	10.29
114	0101	3004	8.43	1401	5201	8.30	8.36
115	0201	2301	10.49	1302	4101	8.25	9.37
116	03173	3201	5.65	3801	4002	7.76	6.71
118	24104	3201	6.18	2707	5501	8.30	7.24
119	2402	3253	7.04	1501	4403	8.10	7.57
120	0101	0201	10.50	5101	5701	7.33	8.92
121	0201	3201	10.29	4406	4406	0.00	5.15
122	2902	6608	5.53	0801	3508	6.96	6.24
123	0301	0302	1.29	3502	5101	5.51	3.40
125	0301	0301	0.00	0702	3508	8.45	4.22
126	2402	2902	10.20	1401	4403	11.17	10.68
127	3077	6901	7.69	5101	5301	3.45	5.57
128	0172	0201	9.49	1362	1401	8.86	9.17
129	2402	3301	10.80	1402	5235	8.11	9.46
131	0301	2601	4.67	07350	44158	12.81	8.74
132	0201	6833	5.20	3801	5001	9.00	7.10
133	0201	6801	5.20	1801	5101	8.73	6.97
134	1101	2402	10.94	0702	3501	9.01	9.98
136	0201	2402	10.66	3503	4901	8.31	9.48
137	2301	2301	0.00	1402	1501	6.99	3.49
138	0205	2402	10.75	3801	5001	9.00	9.88
139	0201	3101	7.52	1401	5107	8.07	7.79
140	2902	3201	6.07	1501	4403	8.10	7.08
141	2601	3201	7.60	4901	5501	9.06	8.33
142	0101	0301	5.75	5701	5301	4.77	5.26
143	0208	11199	7.78	4403	5701	8.59	8.19
144	0201	6801	5.20	3503	4405	7.87	6.54
145	0301	2601	4.67	44225	5174	8.08	6.38
147	0101	0301	5.75	1801	4101	6.80	6.27
148	0201	1101	8.10	1302	4484	7.30	7.70
149	0101	0301	5.75	1301	35235	7.96	6.85

Abbreviations: HED, HLA Evolutionary Divergence; HLA, Human Leukocyte Antigen.

Supplemental Table 5. Cox regression analyses of LMR, NLR and SII indexes for OS and PFS.

	OS			PFS		
**Variable**	**HR**	**95% CI**	**p-value**	**HR**	**95% CI**	**p-value**
Before RHRT						
LMR	0.584	0.374-0.911	0.018	0.673	0.452-1.001	0.051
NLR	1.019	0.897-1.157	0.771	0.953	0.687-1.323	0.776
SII	1.001	0.999-1.000	0.610	0.999	0.999-1.000	0.832
After RHRT						
LMR	0.837	0.702-9.971	0.888	0.638	0.020-19.913	0.798
NLR	1.003	0.978-1.029	0.777	1.003	0.968-1.040	0.872
SII	1.000	0.999-1.000	0.858	1.000	0.999-1.000	0.795

Abbreviations: LMR, Lymphocyte-to-Monocyt Ratio; NLR, Neutrophil-to-Lymphocyte Ratio; SII, Systemic Immune-inflammation Index; HR, Hazard Ratio; CI, Confidence Interval; OS, Overall Survival; PFS, Progression Free Survival; RHRT, Radical Hemithoracic Radiation Therapy

Supplemental Table 6. Mean fluorescence intensity (MFI) variation of PD1+ cells before and after RHRT.

	**Before RHRT** Median (IQR)	**After RHRT** Median (IQR)	**1 month after RHRT** Median (IQR)
**$CD3^+PD1^+$**	428.5 (380.5-461.5)	561.0 (480.3-626.5)	506 (440.5-597.0)
p value#	*<0.00001**		*0.328*
p value##	*0.027**		
**$CD4^+PD1^+$**	393.5 (355.5-431.5)	507.5 (436.0-585.5)	629.5 (529.3-745.5)
p value#	*<0.00001**		*0.053*
p value##	*<0.00001**		
**$CD8^+PD1^+$**	404.0 (356.0-453.3)	517.5 (468.0-589.8)	424.5 (381.8-516.8)
p value#	*<0.00001**		*0.063*
p value##	*0.486*		

# p- value refers to the comparison before RHRT versus after RHRT, and after RHRT versus 1 month after RHRT; ##p value refers to the comparison before RHRT versus 1 month after RHRT; * p value<0.05. *Abbreviations*: IQR, Interquartile Range; PD, Programmed Death; RHRT, Radical Hemithoracic Radiation Therapy;

Supplemental Table 7. Monitoring of Ki67 positivity in T cells based on PD1 expression before and after RHRT.

	**Before RHRT** Median (IQR)	**After RHRT** Median (IQR)	**1 month after RHRT** Median (IQR)
**T cells**			
**Ki67$^+$ PD1$^-$**	1.37 (1.09-1.73)	6.17 (3.38-8.77)	5.06 (1.61-7.15)
p value$^{\#}$	*<0.001**		*0.575*
p value$^{\#\#}$	*0.037**		
**Ki67$^+$ PD1$^+$**	3.11 (2.22-3.73)	19.40 (11.00-24.85)	9.38 (4.28-15.23)
p value$^{\#}$	*<0.001**		*0.093*
p value$^{\#\#}$	*0.022**		
**Th cells**			
**Ki67$^+$ PD1$^-$**	0.92 (0.71-1.18)	2.39 (1.11-3.19)	2.62 (1.62-3.79)
p value$^{\#}$	*<0.001**		*0.646*
p value$^{\#\#}$	*0.012**		
**Ki67$^+$ PD1$^+$**	3.4 (2.43-3.94)	14.70 (8.21-19.80)	13.70 (7.82-15.83)
p value$^{\#}$	*<0.001**		*0.803*
p value$^{\#\#}$	*0.005**		
**CTLs**			
**Ki67$^+$ PD1$^-$**	1.78 (1.16-2.35)	8.89 (3.70-17.30)	5.65 (1.52-8.88)
p value$^{\#}$	*<0.001**		*0.384*
p value$^{\#\#}$	*0.075*		
**Ki67$^+$ PD1$^+$**	2.53 (1.41-3.70)	27.4 (10.00-39.25)	9.59 (3.57-13.95)
p value$^{\#}$	*<0.001**		*0.103*
p value$^{\#\#}$	*0.029**		

# p- value refers to the comparison before RHRT versus after RHRT, and after RHRT versus 1 month after RHRT; ##p value refers to the comparison before RHRT versus 1 month after RHRT; * p value<0.05 . *Abbreviations*: CTLs, Cytotoxic T Lymphocytes; IQR, Interquartile Range; PD-1, Programmed Death 1; RHRT, Radical Hemithoracic Radiation Therapy; Th, T helper.

Supplemental Table 8. Main clinical characteristics of selected MPM patients included in the randomized phase III study

Clinical Parameter	Palliative Arm *(n=22)*	Interventional Arm *(n=21)*
**Age (median, min-max)**	73 (51-84)	70 (33-78)
**Sex (n, %)**		
Male	20 (90)	17 (81)
Female	2 (10)	4 (19)
**Histology (n, %)**		
Epithelioid	16 (73)	20 (95)
Non-epithelioid	6 (27)	1 (5)
**Surgery (n, %)**		
Biopsy	13 (59)	12 (57)
Decortication	3 (14)	4 (19)
Pleurectomy/ decortication	6 (27)	5 (24)

Supplementary Table 9. Percentage of T cell responses specific for MPM-associated antigens in MPM patients before and after RT.

Therapy arm	T cell specificity	Patient number	Percent of T cells positive for at least 1 of the cytokines tested	
			Before RT	1 month after RT
**Palliative arm**	anti-MSTL $CD8^+$	4	0.000	0.000
		26	**0.057**	**0.010**
		30	0.000	0.000
		34	**0.060**	**0.049**
		38	0.000	**0.024**
		49	0.000	0.000
		65	0.000	0.000
		78	0.000	0.000
	anti-MSTL $CD4^+$	1	0.000	0.000
		4	**0.020**	0.000
		6	0.000	0.000
		13	0.000	0.000
		18	0.000	0.000
		26	0.000	0.000
		30	0.000	0.000
		34	0.000	0.000
		35	0.000	0.000
		36	0.000	0.000
		38	**0.039**	0.000
		47	0.000	0.000
		49	0.000	0.000
		61	0.000	0.000
		63	0.000	0.000
		64	0.000	0.000
		67	0.000	0.000
		73	0.000	**0.010**
		76	0.000	0.000
	anti-WT1 $CD8^+$	4	0.000	0.000
		26	0.000	0.000
		30	0.000	0.000
		34	0.000	0.000

		38	0.000	0.000
		49	0.000	0.000
		78	0.000	0.000
	anti-WT1 $CD4^+$	8	0.000	0.000
		18	0.000	0.000
		36	0.000	0.000
		63	0.000	0.000
**Interventional arm**	anti-MSTL $CD8^+$	5	0.000	0.000
		7	0.000	0.000
		19	0.000	0.000
		23	**0.115**	**0.028**
		29	0.000	0.000
		37	**0.102**	**0.064**
		43	0.000	0.000
		53	0.000	0.000
		57	**0.070**	**0.096**
		59	0.000	0.000
	anti-MSTL $CD4^+$	7	0.000	0.000
		15	0.000	0.000
		17	0.000	0.000
		19	0.000	0.000
		20	**0.013**	**0.167**
		22	0.000	0.000
		23	0.000	0.000
		29	0.000	0.000
		31	0.000	0.000
		32	0.000	0.000
		37	**0.038**	**0.090**
		39	0.000	0.000
		43	0.000	0.000
		52	0.000	**0.010**
		53	0.000	0.000
		55	0.000	0.000
		57	0.000	0.000
		59	0.000	**0.036**
		62	0.000	0.000
		77	0.000	0.000
	anti-WT1 $CD8^+$	5	0.000	0.018

	7	0.000	0.000
	19	0.000	0.000
	23	**0.010**	**0.030**
	29	0.000	0.000
	37	**0.161**	**0.108**
	43	0.000	0.000
	53	0.000	0.000
	57	**0.037**	**0.055**
	59	**1.243**	**0.941**
anti-WT1 $CD4^+$	15	0.000	0.000
	20	0.000	**0.184**
	22	0.000	0.000
	29	0.000	**0.195**
	39	0.000	**0.083**
	77	0.000	0.000

Abbreviations: MSTL, mesothelin; RT, radiation therapy; WT-1, Wilms Tumor-1.

Supplemental Table 10. TCR clonality values (Simpson clonality index) obtained before and after RHRT based on age, sex, tumor histology, and tumor site.

	**Before RHRT**<br>Median (IQR)	**After RHRT**<br>Median (IQR)
**Age**		
≤ median	0.04 (0.02-0.06)	0.05 (0.03-0.08)
> median	0.04 (0.04-0.06)	0.05 (0.02-0.08)
p-value	0.25	0.38
**Sex**		
Male	0.05 (0.03-0.06)	0.05 (0.03-0.09)
Female	0.04 (0.03-0.06)	0.05 (0.03-0.07)
p-value	0.39	0.43
**Tumor Histology**		
Epithelioid	0.04 (0.02-0.07)	0.04 (0.02-0.08)
Non-epithelioid	0.05 (0.04-0.05)	0.05 (0.05-0.06)
p-value	0.29	0.35
**Tumor Site**		
Left	0.04 (0.03-0.05)	0.03 (0.01-0.06)
Right	0.04 (0.03-0.06)	0.05 (0.03-0.08)
p-value	0.50	0.20

Abbreviations: IQR, interquartile range; RHRT, radical hemithoracic radiation therapy.

Supplemental Table 11. Association between TCR clonality and HED with OS

	**Chi-squared test**
**Before RHRT**	
TCR clonality	0.739
Median HED	0.381
**After RHRT**	
TCR clonality	0.583
Median HED	0.973

Abbreviations: RHRT, Radical Hemithoracic Radiation Therapy; TCR, T Cell Receptor; HED, HLA Evolutionary Divergence

Supplemental Table 12. SARS-CoV-2-associated templates frequency in samples collected after RHRT

Patient #	SARS-CoV-2 associated TCR sequences	SARS-CoV-2 associated total templates	SARS-CoV-2 associated templates frequency	Frequency (sum) of the 20 most abundant templates measured after RHRT
108	173	252	0.0064	0.0197
110	474	1365	0.0106	0.0244
111	200	317	0.0081	0.0188
116	166	267	0.0057	0.0728
119	296	502	0.0049	0.1604
120	59	82	0.0042	0.1950
121	143	201	0.0061	0.1786
122	192	274	0.0055	0.0341
125	421	778	0.0070	0.2646
126	175	288	0.0042	0.1513
128	499	1320	0.0113	0.0756
129	334	791	0.0068	0.1215
132	143	247	0.0043	0.4170
133	547	1904	0.0138	0.0958
134	287	516	0.0058	0.0996
137	265	819	0.0077	0.2649
140	175	274	0.0070	0.0754
141	100	226	0.0068	0.2670
142	133	313	0.0138	0.0442
148	211	342	0.0084	0.0985
Median (IQR)	196 (59-547)	315 (82-1904)	0.0067 (0.0042-0.0137)	0.0996 (0.0197-0.417)
p value				$p < 0.001$

Abbreviations: IQR, interquartile range; RHRT, radical hemithoracic radiation therapy; SARS-CoV-2, Severe Acute Respiratory Syndrome CoronaVirus 2.

Supplemental Table 13. TCR sequences comparison between our MESORTIBO cohort and patients described in Desai et al (5)

**MESORTIBO cohort (sample obtained at a single time point)**	**Desai et al** (5) **cohort (paired samples before and after ICI therapy)**	**Common sequences found in 3 samples**	**Sequences with a higher frequency (>1.5X) in post-ICI therapy samples**	**Sequences identified in TCR databases**	**Sequences with an unknown specificity**
Meso108 after RHRT	Initiate_35	38	9	5	4
Meso110 before RHRT	Initiate_36	298	215	127	88
Meso110 after RHRT	Initiate_36	26	16	6	10
Meso122 after RHRT	Initiate_18	38	26	11	15
Meso128 before RHRT	Initiate_16	114	10	6	4
Meso133 before RHRT	Initiate_08	1319	229	129	100
Meso133 before RHRT	Initiate_11	1953	147	69	78
Meso133 before RHRT	Initiate_14	483	73	42	31
Meso133 before RHRT	Initiate_16	601	39	20	19
Meso133 before RHRT	Initiate_17	1112	63	42	21
Meso133 before RHRT	Initiate_23	227	196	90	106
Meso133 before RHRT	Initiate_32	490	74	25	49
Meso133 before RHRT	Initiate_35	600	111	67	44
Meso133 before RHRT	Initiate_36	389	268	119	149
Meso133 before RHRT	NivoMes_13	1316	237	116	121
Meso133 before RHRT	NivoMes_22	1207	953	359	594
Meso134 before RHRT	Initiate_18	470	270	108	162
Meso137 before RHRT	Initiate_37	840	76	41	35
Meso140 before RHRT	Initiate_24	614	170	88	82
Meso140 before RHRT	NivoMes_11	394	42	21	21
Total		12,529	3,224	1,491	1,733

Abbreviations: ICI, Immune Checkpoint Inhibitors; TCR, T-Cell receptor; TCR databases: VDJdb [https://vdjdb.cdr3.net/search](6), McPAS-TCR [https://friedmanlab.weizmann.ac.il/McPAS-TCR/](7), and TCR Match [http://tools.iedb.org/tcrmatch/](8).

Figures

Supplemental Figure 1

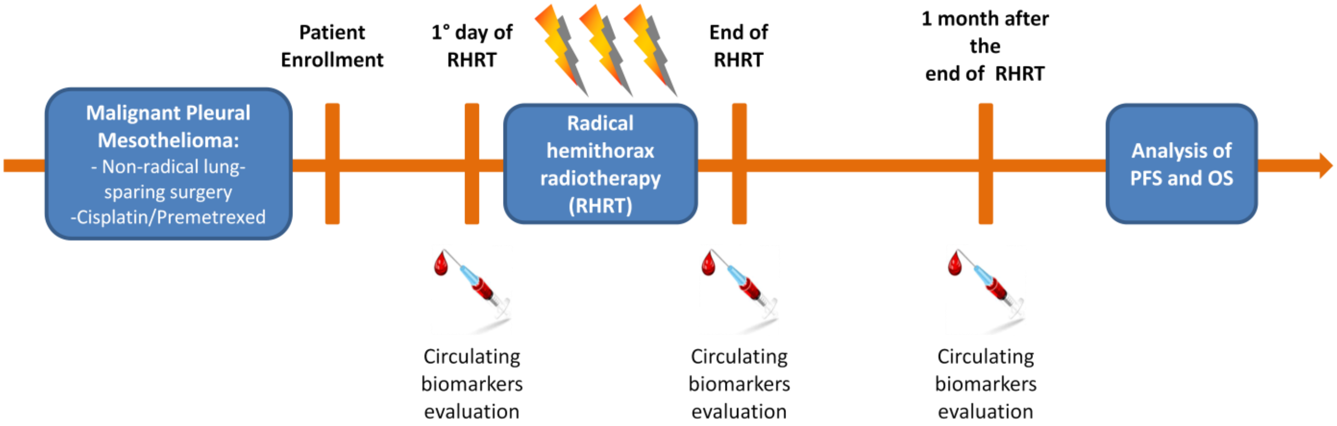

Supplemental Figure 1. Schematic representation of the study design; *Abbreviations:*OS, Overall Survival; PFS, Progression-Free Survival.

Supplemental Figure 2

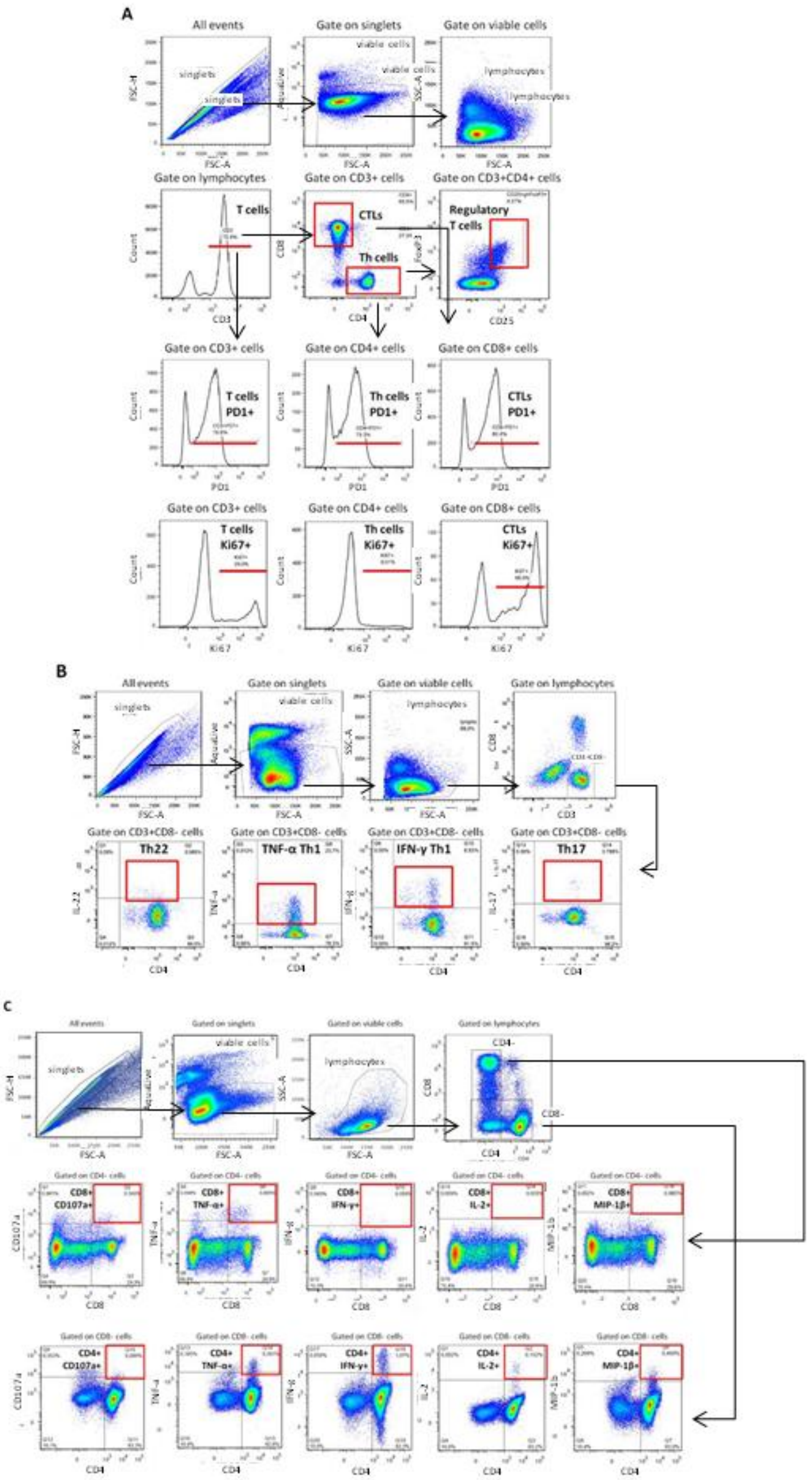

Supplemental Figure 2. Gating strategy for the identification of immune cell populations. A. Identification of T cells, Th cells, CTLs, and regulatory T cells, and their markers of proliferation (Ki67) and inhibition (PD-1). **B.** Identification of Th1 (TNF-α and IFN-γ), Th17 and Th22 cells after PMA and ionomycin stimulation. Th cells were identified as $CD3^{+}CD8^{-}$ cells due to the CD4 downregulation after stimulation. **C.** Definition of $CD8^{+}$ and $CD4^{+}$ T cells producing TNF-α, IFN-γ, IL-2, and/or MIP-1β, or degranulating ($CD107a^{+}$) after peptide stimulation. *Abbreviations:* CTL, cytotoxic T lymphocytes; IFN, interferon; Th, T helper; TNF, tumor necrosis factor; Treg, regulatory T cells.

Supplemental Figure 3

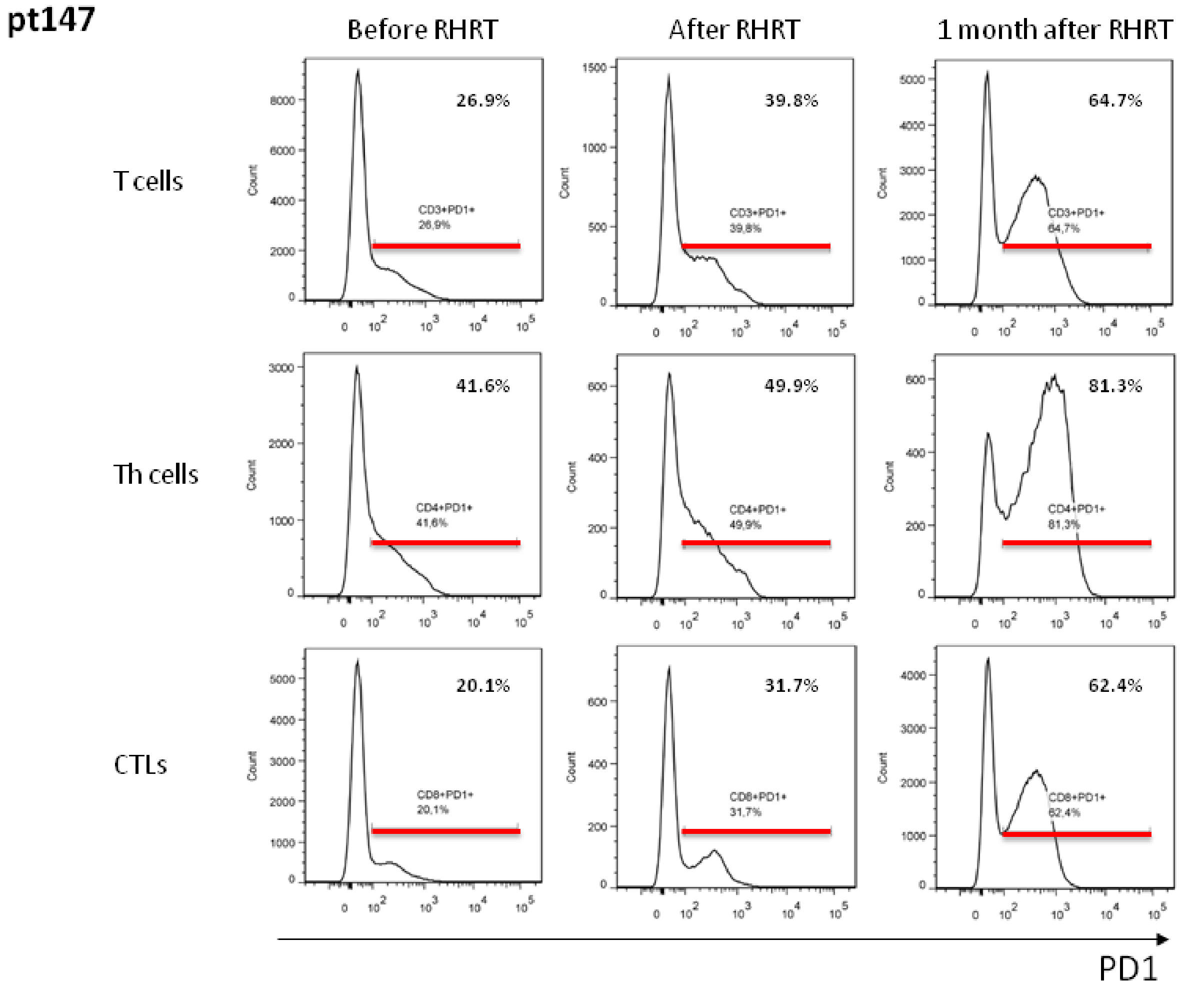

Supplemental Figure 3. Flow cytometry histogram plots depicting variation of PD1 expression on CD3+, CD4+ and CD8+ T cells of patient number 127 before and after RHRT. The percentage of PD1 positive cells is added in each plot. Abbreviations: CTLs, cytotoxic T lymphocytes; pt, patient; PD, programmed death ; RHRT, radical hemithoracic radiotherapy; Th, T helper.

Supplemental Figure 4

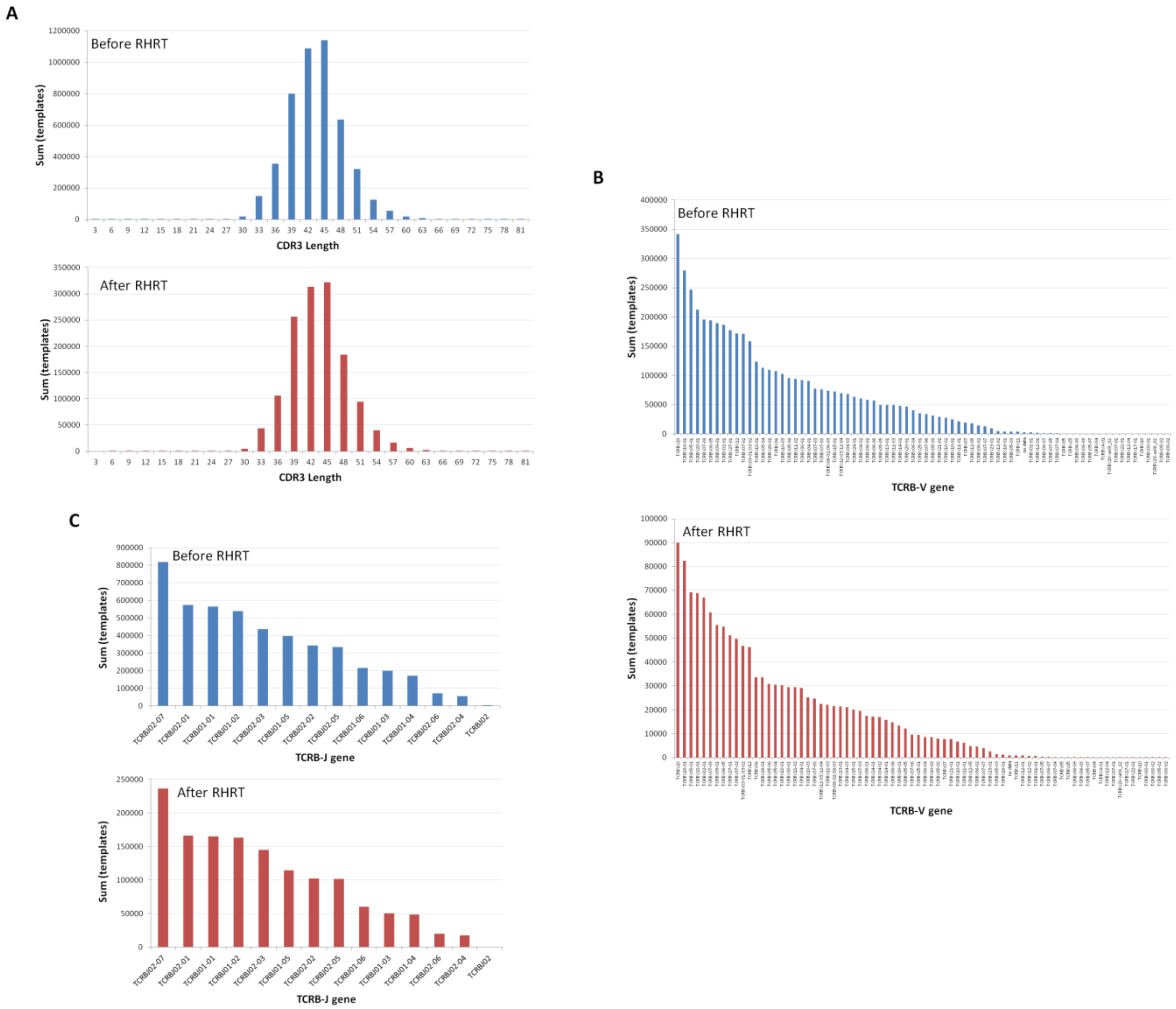

Supplemental Figure 4. Global characteristics of TCR sequences. Data were represented as sum of templates for each CDR3 length, TRBV and TRBJ gene family. **A.** CDR3 lengths distribution in samples obtained before and after RHRT. **B.** TRBV gene families prevalence in samples obtained before and after RHRT. **C.** TRBJ gene families found in samples obtained before and after RHRT.

Supplemental Figure 5

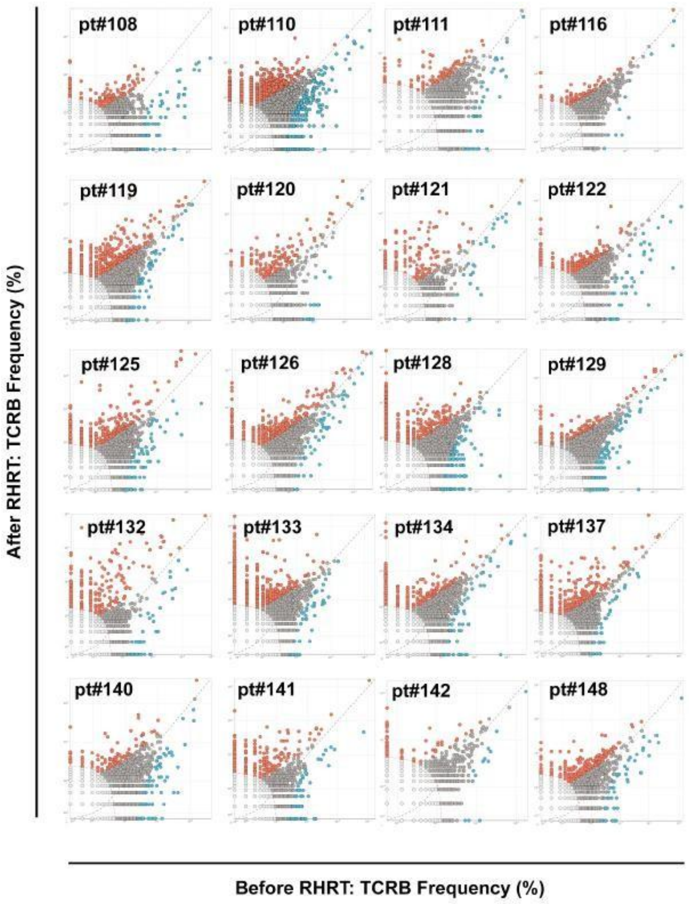

Supplemental Figure 5. Differential abundance of every T-cell clone detected as unique TCR in each patient before and after RHRT. Blue dots represent T-cell clones more abundant before RHRT (contracted clones), red dots represent T-cell clones expanded after RHRT, grey dots are T-cell clones not significantly changed before and after RHRT, white dots are T-cell clones excluded from this analysis. *Abbreviations:* pt, patient; RHRT, radical hemithoracic radiotherapy; TCRB, T-Cell Receptor β chain.

References

1. Thomas AM, Santarsiero LM, Lutz ER, Armstrong TD, Chen Y-C, Huang L-Q, Laheru DA, Goggins M, Hruban RH, Jaffee EM. Mesothelin-specific CD8(+) T cell responses provide evidence of in vivo cross-priming by antigen-presenting cells in vaccinated pancreatic cancer patients. *J Exp Med* (2004) 200:297–306. doi: 10.1084/jem.20031435

2. Chen Y, Ayaru L, Mathew S, Morris E, Pereira SP, Behboudi S. Expansion of anti-mesothelin specific CD4+ and CD8+ T cell responses in patients with pancreatic carcinoma. *PLoS One* (2014) 9:e88133. doi: 10.1371/journal.pone.0088133

3. Oka Y, Elisseeva OA, Tsuboi A, Ogawa H, Tamaki H, Li H, Oji Y, Kim EH, Soma T, Asada M,

et al. Human cytotoxic T-lymphocyte responses specific for peptides of the wild-type Wilms' tumor gene (WT1) product. *Immunogenetics* (2000) 51:99–107. doi: 10.1007/s002510050018

4. Müller L, Knights A, Pawelec G. Synthetic peptides derived from the Wilms' tumor 1 protein sensitize human T lymphocytes to recognize chronic myelogenous leukemia cells. *Hematol J* (2003) 4:57–66. doi: 10.1038/sj.thj.6200220

5. Desai AP, Kosari F, Disselhorst M, Yin J, Agahi A, Peikert T, Udell J, Johnson SH, Smadbeck J, Murphy S, et al. Dynamics and survival associations of T cell receptor clusters in patients with pleural mesothelioma treated with immunotherapy. *J Immunother Cancer* (2023) 11:e006035. doi: 10.1136/jitc-2022-006035

6. Goncharov M, Bagaev D, Shcherbinin D, Zvyagin I, Bolotin D, Thomas PG, Minervina AA, Pogorelyy MV, Ladell K, McLaren JE, et al. VDJdb in the pandemic era: a compendium of T cell receptors specific for SARS-CoV-2. *Nat Methods* (2022) 19:1017–1019. doi: 10.1038/s41592-022-01578-0

7. Tickotsky N, Sagiv T, Prilusky J, Shifrut E, Friedman N. McPAS-TCR: a manually curated catalogue of pathology-associated T cell receptor sequences. *Bioinformatics* (2017) 33:2924–2929. doi: 10.1093/bioinformatics/btx286

8. Chronister WD, Crinklaw A, Mahajan S, Vita R, Koşaloğlu-Yalçın Z, Yan Z, Greenbaum JA, Jessen LE, Nielsen M, Christley S, et al. TCRMatch: Predicting T-Cell Receptor Specificity Based on Sequence Similarity to Previously Characterized Receptors. *Front Immunol* (2021) 12:640725. doi: 10.3389/fimmu.2021.640725
